# The tiger beetles (Coleoptera, Carabidae, Cicindelinae) of Israel and adjacent lands

**DOI:** 10.3897/zookeys.578.7383

**Published:** 2016-04-08

**Authors:** Andrey V. Matalin, Vladimir I. Chikatunov

**Affiliations:** 1 Education-Scientific Centre Ecology & Biodiversity, Moscow State Pedagogical University, Moscow 129164, Russia; 2 Department of Biology, Russian National Research Medical University named after N.I. Pirogov, Moscow 117997, Russia; 3 Department of Zoology, Tel-Aviv University, Tel Aviv 69978, Israel

**Keywords:** Carabidae, tiger beetles, Cicindelinae, Israel, Lebanon, Jordan, Syria, Egypt, Sinai, Levant, Mediterranean, fauna, endemic, near-endemic, catalogue, key, distribution, phenology, faunogenesis

## Abstract

Based on field studies, museums collections and literature sources, the current knowledge of the tiger beetle fauna of Israel and adjacent lands is presented. In Israel eight species occur, one of them with two subspecies, while in the Sinai Peninsula nine species of tiger beetles are now known. In the combined regions seven genera from two tribes were found. The Rift Valley with six cicindelids species is the most specious region of Israel. *Cylindera contorta valdenbergi* and *Cicindela javeti azari* have localized distributions and should be considered regional endemics. A similarity analysis of the tiger beetles faunas of different regions of Israel and the Sinai Peninsula reveal two clusters of species. The first includes the Great Rift Valley and most parts of the Sinai Peninsula, and the second incorporates most regions of Israel together with Central Sinai Foothills. Five distinct adult phenological groups of tiger beetles can be distinguished in these two clusters: active all-year (three species), spring-fall (five species), summer (two species), spring-summer (one species) and spring (one species). The likely origins of the tiger beetle fauna of this area are presented. An annotated list and illustrated identification key of the Cicindelinae of Israel and adjacent lands are provided.

## Introduction

The first data about tiger beetles of Palestine were published in the first third of the XXth century. In 1913 Sahlberg described from Wadi El Nawaime (modern Wadi en Nu’eima) *Cicindela littoralis aulicoides*. In 1934 Mandl recorded for the Palestine two subspecies of *Cicindela littoralis*: *Cicindela littoralis winkleri* and *Cicindela littoralis aulicoides*. The first species list of Palestinian Coleoptera including five species of tiger beetles was published by Bodenheimer in 1937. Around the same time, the first information about cicindelids of the Sinai Peninsula appeared and *Cicindela aulica* (Horn, 1931), *Cicindela littoralis aulicoides* (Mandl, 1934) and *Megacephala euphratica* (Schatzmayr, 1936) were recorded. Unfortunately, detailed locality data and collecting dates for specimens of these species were often incomplete.

A second wave of tiger beetles studies in the Levant was completed in the last third of XXth century. Alfieri (1976) published the catalogue of Egyptian Coleoptera with information about 11 species of tiger beetles, six of which were recorded for the Sinai Peninsula. The first data about Cicindelinae of Israel were published by Valdenberg (1983, 1985) and Nussbaum (1987). It should be noted that these papers also contained information about tiger beetles of the Sinai Peninsula. In all eight species were recorded from Israel and seven species for the Sinai. Unfortunately, in the paper by Nussbaum (1987) data about localities for several species given in the text and on the maps do not coincide.

Since the beginning of 2000 interest in the Cicindelinae of the Middle East has increased significantly (El-Moursy et al. 2001; Franzen 2001, 2007; Finkel et al. 2002; Wiesner 2002, 2005; Abdel-Dayem et al. 2003; Rittner 2003; Abdel-Dayem 2004, 2012; Chikatunov et al. 2006; Avgin and Özdikmen 2007; Franzen 2007; Avgin and Wiesner 2009; Ptashkovsky 2009; Deuve 2011, 2012; Abdel-Dayem and Kippenhan 2013; Jaskuła and Rewicz 2014). These studies revealed the presence of several species of tiger beetle previously unknown from the area. For example, *Habrodera nilotica* (Dejean, 1825), *Hypaetha singularis* (Chaudoir, 1876) and *Cephalota littorea* (Forskål, 1775) were recorded for the first time in Israel (Chikatunov et al. 2006). However, in the next publications these species were not included (Ptashkovsky 2009).

During the last decade, new information about the distribution of tiger beetles in different parts of the Levant has accumulated, and we include these new records here.

## Material and methods

Specimens and data for this report come from the following museums and private collections:

TAU
Tel Aviv University (Israel) 


ZMUM
Zoological Museum of Moscow State University (Moscow, Russia) 


MPU
Moscow State Pedagogical University (Moscow, Russia) 


SIZ
 I.I. Schmalhausen Institute of Zoology, National Academy of Sciences of Ukraine (Kiev, Ukraine) 


cJW
 collection of Jürgen Wiesner (Wolfsburg, Germany) 


cIOv
 collection of Igor’ Ovsyannikov (Moscow, Russia) 


The nomenclature of elytral pattern follows Acciavatti and Pearson (1989); the nomenclature of male internal sac follows Matalin (1998); the chorology follows Vigna Taglianti et al. (1999) with some additions; the regions of Israel and the Sinai Peninsula (Egypt) follow Nussbaum (1987). The similarity of the faunas of tiger beetles was calculated using complete linkage procedure (squared Euclidean distances).

The species included here that are not yet recorded from Israel are marked in the catalogue and in the key with a symbol (○).

## Results and discussion

### Catalogue of the tiger beetles of Israel and adjacent lands

#### Family Carabidae Latreille, 1802

##### Subfamily Cicindelinae Latreille, 1802

###### Tribe Cicindelini Latreille, 1802

####### Subtribe Cicindelina Latreille, 1802

######## Genus *Calomera* Motschulsky, 1862

######### Calomera aulica aulica

Taxon classificationAnimaliaColeopteraCarabidae

(Dejean, 1831)

########## General distribution.

**Europe** Greece; **Asia** Lebanon, Israel, Jordan, Syria, Egypt (Sinai), Saudi Arabia, Arab Emirates, Oman, Yemen, Bahrain, Iran, Iraq, Pakistan; **Africa**: Cape Verde Islands, Senegal, Guinea Bissau, Mauritania, Morocco, Tunisia, Algeria, Libya, Sudan, Chad, Egypt, Somalia, Eritrea, Djibouti.

########## References.

**Israel** – Bodenheimer 1937: 108 (as *Cicindela*); Valdenberg 1983: 43, 46 (as *Cicindela*), 1985: 37 (as *Cicindela*); Cassola 1985: 56 (as *Lophyridia*); Nussbaum 1987: 9-10 (as *Cicindela*); Wiesner 1992: 151 (as *Lophyridia*); Puchkov and Matalin 2003: 99; Rittner 2003 (as *Lophyridia*); Ptashkovsky 2009: 8-9 (as *Lophyra*); **Egypt (Sinai)** – Horn 1931: 162 (as *Cicindela*); Alfieri 1976: 1 (as *Cicindela*); Cassola 1984: 56 (as *Lophyridia*); Nussbaum 1987: 9-10 (as *Cicindela*); Wiesner 1992: 151 (as *Lophyridia*); Werner 2000: 98 (as *Lophyridia*); El-Moursy et al. 2001: 66 (as *Cicindela*); Abdel-Dayem et al. 2003: 205 (as *Lophyridia*); Puchkov and Matalin 2003: 103; Abdel-Dayem 2004: 74 (as *Lophyridia*).

########## Distribution

**Figure 1.**
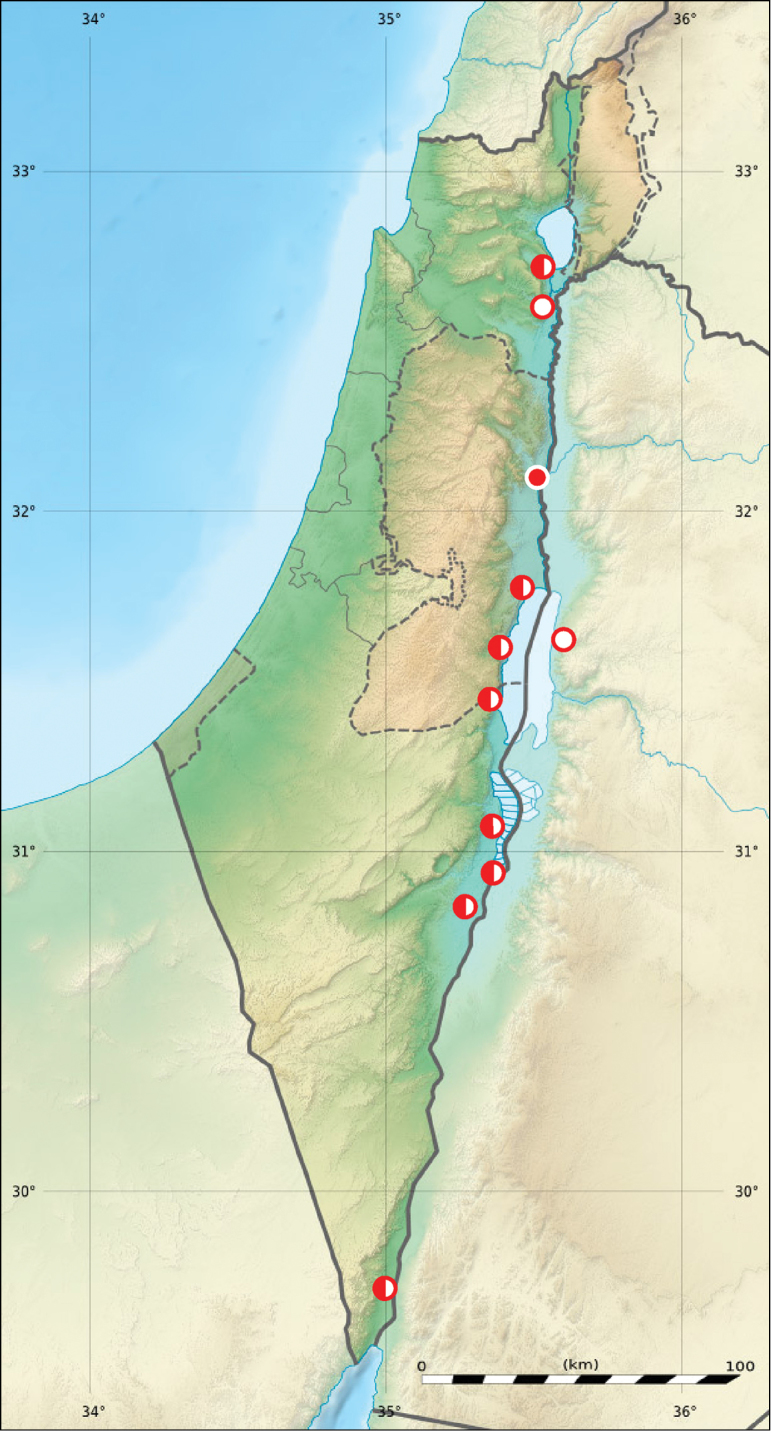
Distribution of *Calomera aulica aulica* in Israel, Palestine and border areas of Jordan (open circles records before year 1949, half-solid circles records between years 1950–1999, solid circles records after year 2000; map source Eric Gaba Wikimedia Commons user: Sting and Wikimedia Commons user: NordNordWest, URL https://upload.wikimedia.org/wikipedia/commons/7/7c/Israel_relief_location_map.jpg)

**Figure 2.**
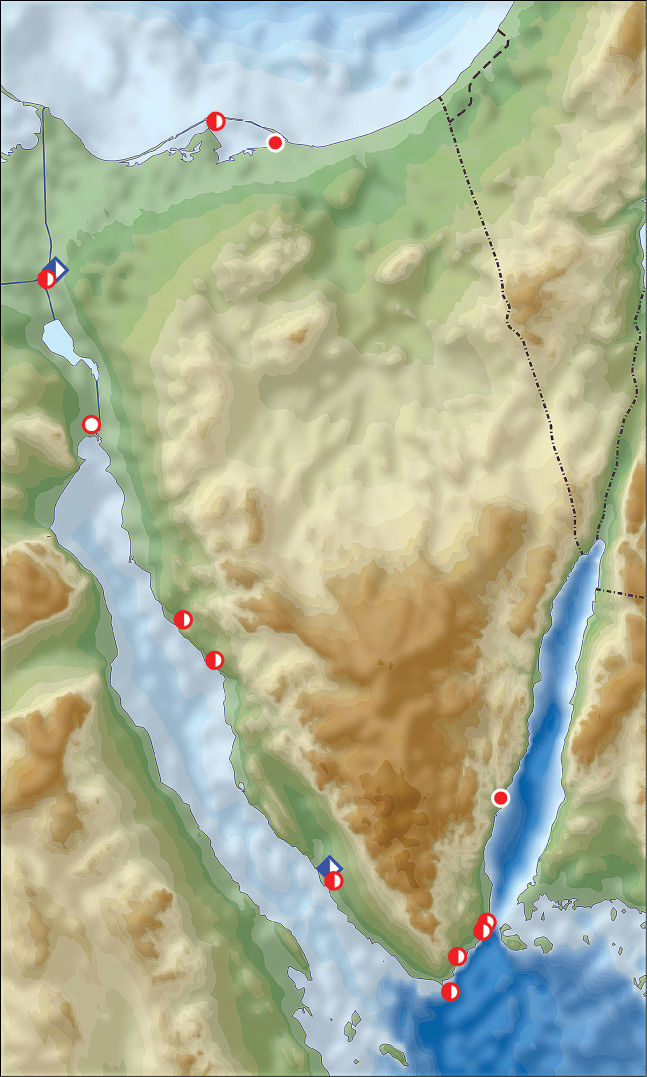
Distribution of *Calomera aulica aulica* (red circles) and *Calomera littoralis aulicoides* (blue rhombs) in Sinai Peninsula, Egypt (open symbols records before year 1949, half-solid symbols between years 1950–1999, solid symbols records after year 2000; URL map source https://upload.wikimedia.org/wikipedia/commons/5/59/Sinai_relief_location_map.svg).

(Figs 1, 2). **Israel (including State of Palestine), Jordan Valley**: *Zor Deir Shaman*, 15.III.2005, I. Zonstein - 1♀; *Gesher*, 17.VIII.1939, H. Bytinski-Salz 1♂ (both TAU); *Kinneret zone* (after Nussbaum 1987); **Dead Sea Area**: *Ne`ot HaKikkar*, 7.V.1980, leg. A. Valdenberg 5♂♂ 7♀♀; 16.VII.1999, 13.VIII.1999, 11.IX.1999, and 12.XII. 1999, light trap BL, leg. I. Yarom & V. Kravchenko 2♂♂ 7♀♀; *Sedom*, 15.VIII.1957, leg. J. Wahrman 1♂ 2♀♀ (all TAU); ‘*En Gedi*, 19-29.V.1989, leg. G. Müler 1♀; *Qalya*, 28.VIII.1986, 28.6.1987, leg. Y. Nussbaum 2♂♂ (both cJW); ‘*Enot Qane* (after Nussbaum 1987); **Arava Valley**: *Be`er Ora*, 3.IV.1997, leg. V. Chikatunov 3♂♂ 1♀; ‘*En ‘Iddan*, 15.VII.1999, leg. I. Yarom & V. Kravchenko 1♂ 1♀ (all TAU).

**Jordan, Ma’Daba**: *Callirhoe*, 7.VI.1942, leg. H. Bytinski-Salz 2♀ (TAU).

**Egypt (Sinai), Northern Sinai**: *Sabkhat al Bardawil*, 25.VIII.1967, leg. I. Margalit 3♀♀; 24.VIII.1979, leg. A. Valdenberg 1♂ 2♀♀ (TAU); *Ismailia* (after Alfieri 1976); *Zaranik Protectorate* (after El-Moursy et al. 2001; Abdel-Dayem et al. 2003; Abdel-Dayem 2004); **Sinai Mountains**: *20 km NE of Dahab*, saline land, 4.VIII.2008, leg. A. Sokolov 4♂♂5♀♀ (MPU); **Southwestern Sinai**: *Suez* – 1♂ (ZMUM); *Nabeq*, 17.VIII.1971, leg. J. Kugler 1♂ 2♀♀; 8.V.1980, leg. A. Valdenberg 4♂♂ 6♀♀; *Ras al Tantur*, 5.VII.1957, leg. Ch. Lewinsohn 2♂♂ 1♀, 17.VIII.1971, leg. M. Kaplan -1♀ (all TAU); *15 km W Ofira*, Golf v. Elat, Straße von Tiran, 3.IV.1981, leg. G. Gerdes 1♂ (cJW); *Wadi Gharandal*, 20.V.1969, leg. Tsabar 1♂ (TAU); *Abu Zenima*, *Wadi Tayebeh* (both after Alfieri 1976); *El Tor* (after Alfieri 1976; Abdel-Dayem et al. 2003; Abdel-Dayem 2004); *Ras Muhammad* (after Nussbaum 1987).

######### Calomera littoralis aulicoides

Taxon classificationAnimaliaColeopteraCarabidae

(J. Sahlberg, 1913)

########## General distribution.

**Asia** Turkey, Israel, Jordan, Syria, Egypt (Sinai), Saudi Arabia, Iran, Iraq; **Africa** Egypt.

########## References.

**Israel** – Sahlberg 1913: 3 (as *Cicindela*); Mandl 1934: 244-245 (as *Cicindela lunulata nemoralis aulicoides*), 1982: 93-94 (as *Lophyridia aulicoides*); Valdenberg 1983: 44, 47 (as *Cicindela*), 1985: 36 (as *Cicindela*); Nussbaum 1987: 11-12 (as *Cicindela*); Wiesner 1992: 149 (as *Lophyridia*); Puchkov and Matalin 2003: 100; Chikatunov et al. 2006: 293; **Egypt (Sinai)** Mandl 1934: 244-245 (as *Cicindela lunulata nemoralis aulicoides*), 1982: 94 (as *Lophyridia aulicoides*); Alfieri 1976: 2 (as *Cicindela lunulata aulicoides*); Wiesner 1992: 149 (as *Lophyridia*); Abdel-Dayem et al. 2003: 207 (as *Lophyridia*); Puchkov and Matalin 2003: 103; Abdel-Dayem 2004: 74 (as *Lophyridia*).

########## Distribution

**Figure 3.**
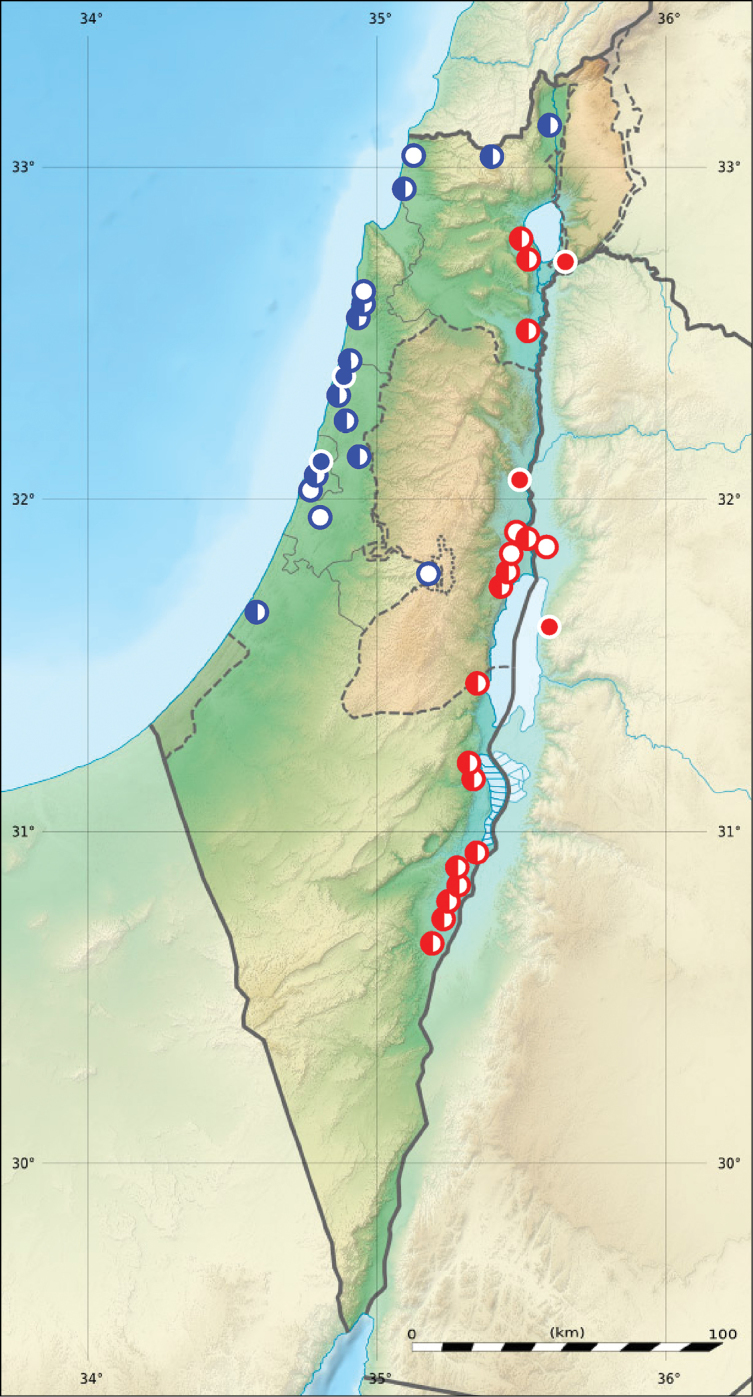
Distribution of two subspecies of *Calomera littoralis* in Israel, Palestine and border areas of Jordan (red circles – *Cicindela littoralis aulicoides*, blue circles – *Calomera littoralis winkleri*, open circles records before year 1949, half-solid circles – records between years 1950–1999, solid circles – records after year 2000; map source Eric Gaba Wikimedia Commons user: Sting and Wikimedia Commons user: NordNordWest, URL https://upload.wikimedia.org/wikipedia/commons/7/7c/Israel_relief_location_map.jpg).

(Figs 2–3). **Israel (including State of Palestine), Golan Heights**: *Hammat Gader*, 2.X.2002, leg. V. Kravchenko & V. Chikatunov 1♀ (TAU); **Lower Galilee**: *Teverya*, 16.VI.1981, leg. A. Valdenberg 1♀; *Kinneret*, 16.VI.1981, leg. A. Valdenberg 3♂♂ 3♀♀; **Jordan Valley**: *Zor Deir Shaman*, Yarden bank, 32°02'30’'N, 35°30'E, 15.III.2005, leg. L. Friedman & I. Zonstein 3♂♂ 1♀ (TAU); *Allenby bridge* (after Mandl 1982); *Tomer, Ma’oz-Hayyim* (both after Nussbaum 1987); **Dead Sea Area**: *Yeriho*, Jordan, Palestine, 24.IV.27 – 1♀; ‘*Enot Zuqim*, 13.III.1993, leg. V. Chikatunov 2♂♂ 1♀, 9.VI.1997, leg. L. Friedman 1♂, 1.II.1994, and 13.III.1994, leg. V. Chikatunov 3♂♂ 2♀♀; *Ne`ot HaKikkar*, 19.IV.1999, 16.VII.1999, 13.VIII.1999, and 11.IX.1999, leg. I. Yarom & V. Kravchenko 17♂♂ 11♀♀; *Sedom*, 19.VIII.1957, J. Wahrman - 2♂♂; *Qalya*, 9.VI.1981, leg. A. Valdenberg 4♂♂ 6♀♀ (all TAU); ‘*En Gedi*, 1-13.V.1980, 19-29.V.1989, leg. G. Müller 3♀♀; *Newe Zohar*, 24.VI.1987, leg. Y. Nussbaum 1♂ (both cJW); *Wadi El Nawaime* [*Wadi en Nu’eima*] (after Sahlberg 1913); *Enot Qane* (after Nussbaum 1987); **Arava Valley**: ‘*En Zin*, 30°53.60'N, 35°09.17'E, light trap BL, 12.X.1999, leg. I. Yarom & V. Kravchenko 1♂; *Hazeva*, field school, 30°46.70'N, 35°14.25'E, light trap BL, 20.III.1999, 21.V.1999, leg. I. Yarom & V. Kravchenko 1♂ 3♀♀; ‘*En ‘Iddan*, 20.VI.1995, leg. I. Yarom & A. Freidberg 2♂♂ 1♀; 15.VII.1999, leg. I. Yarom & V. Kravchenko – 3♂♂ 4♀♀; *Nahal Shezaf*, 18.V.1999, 8.VI.1999, light trap, leg. I. Yarom & V. Kravchenko 1♂ 1♀; *Nahal Neqarot*, 10.III.1999, leg. I. Yarom & V. Kravchenko 1♀ (all TAU).

**Jordan, Al Balqā**’: *Al Maghtas*, 12.II.1942, leg. H. Bytinski-Salz 1♂ 2♀♀(TAU); **Ma’Dabā**: *Suwayma*, Dead Sea, 5.IV.2000, leg. G. & I. Zappi 1♂1♀ (MPU).

**Egypt (Sinai), Northern Sinai**: *Ismailia* (after Alfieri 1976); **Southwestern Sinai**: *El Tor* (after Alfieri 1976; Abdel-Dayem et al. 2003; Abdel-Dayem 2004).

######### Calomera littoralis winkleri

Taxon classificationAnimaliaColeopteraCarabidae

(Mandl, 1934)

########## General distribution.

**Europe** Greece, Armenia, Azerbaijan; **Asia** Cyprus, Turkey, Lebanon, Israel, Jordan, Syria, Iran, Iraq, Afghanistan, Turkmenistan.

########## References.

**Israel** – Mandl 1934: 240, 243, 245 (as *Cicindela*); Naviaux 1983: 82 (as *Lophyridia*), Valdenberg 1983: 44, 46 (as *Cicindela*), 1985: 36 (as *Cicindela*); Nussbaum 1987: 11, 13 (as *Cicindela*); Wiesner 1992: 151 (as *Lophyridia*); Puchkov and Matalin 2003: 101; Rittner 2003 (as *Cicindela*); Chikatunov et al. 2006: 293; Ptashkovsky 2009: 8-9 (as *Cicindela*).

########## Distribution

(Fig. 3). **Israel, Upper Galilee**: *Hula*, 23.VI.1952, leg. J. Wahrman 5♂♂ 4♀♀; 8.III.1976, leg. M. Kaplan - 1♂; *Sasa*, 18.III.1951, leg. J. Wahrman 1♀ (all TAU); **Northern Coastal Plain**: ‘*Akko*, 7.VIII.1980, leg. A. Valdenberg 3♂♂ 4♀♀; *Ma’agan Mikha`el*, 17-18.III.1979, 27.VI.1979, 26.III.1980, 24.IV.1980, 24.VI.1980, leg. A. Valdenberg 17♂♂ 28♀♀; 4.VI.1983, leg. E. Sney-Dor 2♂♂ 1♀; *Nahariyya*, 19.VI.1942, leg. H. Bytinski-Salz 1♂ 2♀♀; *Dor*, Horvat Tantura, sea-shore, 13.IX.1949, leg. J. Wahrman 1♀; *Zikhron Ya’aqov*, 29.VI.1998, leg. A. Traub 3♀♀ (all TAU); **Central Coastal Plain**: *Hadera*, 28.III.2008, leg. G. Wizen - 1♂ 1♀; *Bat Yam*, 13.VII.1945, leg. H. Bytinski-Salz 1♂ 3♀♀; *Hofit*, 21.IX.1994, leg. F. Kaplan & A. Freidberg 1♀; *Mishmeret*, 3.VIII.1983, leg. A. Freidberg 2♂♂ 3♀♀; *Qesarya*, 11.VII.1979, and 10.VIII.1979, leg. A. Valdenberg 3♂♂ 4♀♀; *Tel Aviv*, 20.VI.1982, leg. A. Valdenberg 1♂ 2♀♀; 12.IV.2003, leg. V. Kravchenko & V. Chikatunov 4♂♂ 2♀♀; *Rosh Ha’Ayin*, 16.IV.1993, leg. A. Freidberg & F. Kaplan 1♂ 1♀; *Rehovot*, 5.V.1942, leg. H. Bytinski-Salz 3♂♂ 2♀♀ (all TAU); **Southern Coastal Plain**: *Nitzanim*, 13.VII.1981, leg. A. Valdenberg 3♂♂ 2♀♀ (TAU); **Judean Hills**: Jerusalem 1♂ (after Mandl 1934: 40, Fig. 65).

######## Genus *Cephalota* Dokhtouroff, 1883

######### Cephalota (Taenidia) zarudniana vartianorum

Taxon classificationAnimaliaColeopteraCarabidae

(Mandl, 1967)

########## General distribution.

**Asia** Israel, Syria, Iran, Iraq.

########## References.

**Israel** – Naviaux 1983: 78; Valdenberg 1983: 43 (as *Cicindela jarudniana vartinorum*), 45 (as *Cicindela jorudniana*), 1985: 37 (as *Cicindela jarudniana*); Nussbaum 1987: 9, 13 (as *Cicindela jarudniana*); Wiesner 1992: 177; Puchkov and Matalin 2003: 103; Chikatunov et al. 2006: 293 (as *Cephalota deserticola*); Ptashkovsky 2009: 8-9 (as *Cephalota deserticola*).

########## Distribution

**Figure 4.**
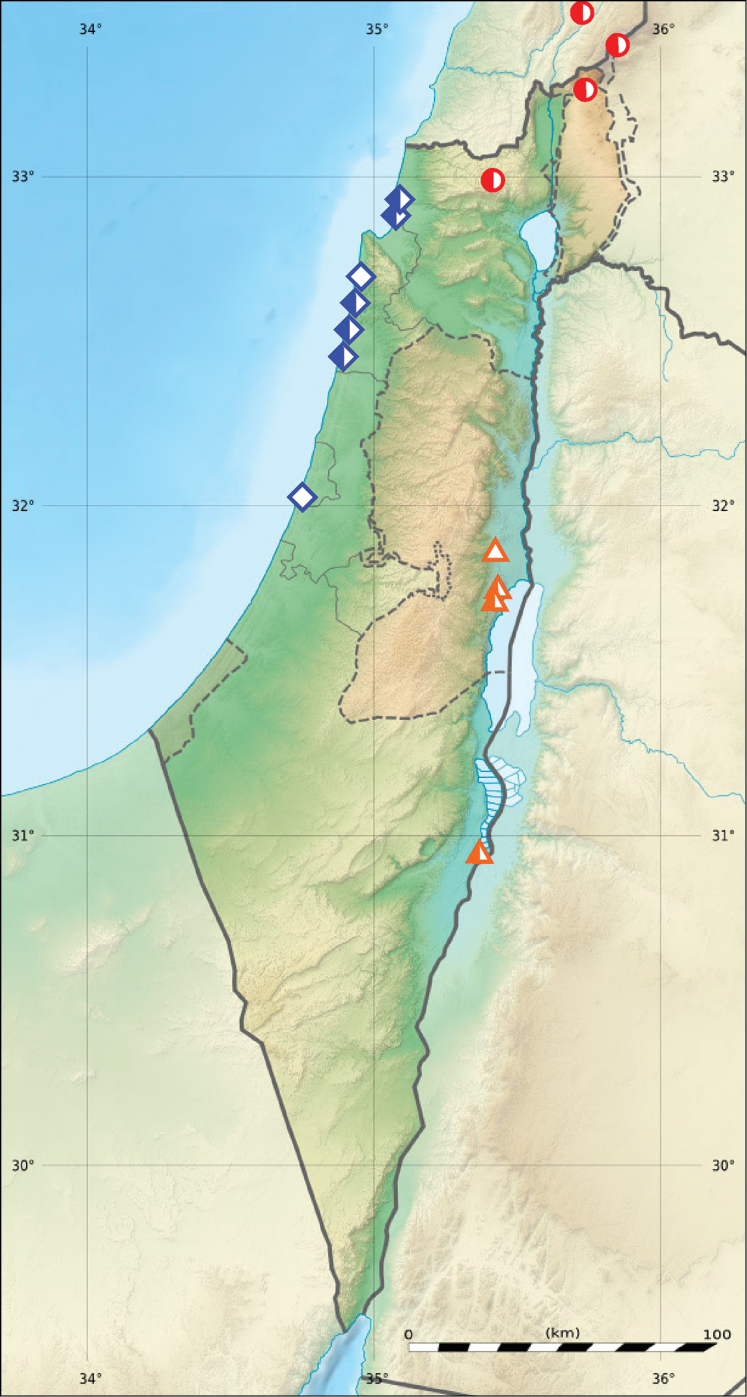
Distribution of *Cephalota zarudniana vartianorum* (orange triangles), *Cicindela javeti azari* (red circles) and *Cylindera contorta valdenbergi* (blue rhombs) in Israel, Palestine and border areas of Lebanon (open symbols records before year 1949, half-solid symbols – records between years 1950–1999; map source Eric Gaba Wikimedia Commons user: Sting and Wikimedia Commons user: NordNordWest, URL https://upload.wikimedia.org/wikipedia/commons/7/7c/Israel_relief_location_map.jpg).

(Fig. 4). **Israel (including State of Palestine), Dead Sea Area**: *Yeriho*, 24.IV.1927, leg. O. Theodor 1♂; ‘*Enot Zuqim*, 1.II.1994, and 13.III.1994, leg. V. Chikatunov 1♂ 2♀♀; *Qalya*, 6.V.1980, leg. A. Valdenberg 2♂♂ 3♀♀; *Ne`ot HaKikkar*, 7.V.1980, leg. A. Valdenberg 4♂♂; 28.IV.1984, leg. E. Shney-Dor 2♀♀; 19.IV.1999, leg. I. Yarom & V. Kravchenko 1♂1♀ (all TAU), 7.V.1980, leg. R. Naviaux 1♀; V.1990, leg. Orbach 1♂1♀ (both cJW).

########## Comments.

References to *Cephalota zarudniana vartianorum* (Mandl, 1967) as *Cephalota deserticola* (Faldermann, 1836) (Chikatunov et al. 2006; Ptashkovsky 2009) were based on two mis-identified males from Qalya by K. Mandl. According to Franzen and Wiesner (1998) *Cephalota deserticola* is distributed in the western part of Central Asia, as well as in Iran, Azerbaijan, Armenia and north-eastern Turkey.

######### (○) Cephalota (Taenidia) littorea littorea

Taxon classificationAnimaliaColeopteraCarabidae

(Forskål, 1775)

########## General distribution.

**Asia** Egypt (Sinai), Saudi Arabia; **Africa** Egypt, Sudan, Eritrea.

########## References.

**Egypt (Sinai)** Forskål 1775: 77 (as *Cicindela*); Alfieri 1976: 2 (as *Cicindela*); Valdenberg 1983: 44, 46 (as *Cicindela*), 1985: 37 (as *Cicindela*); Nussbaum 1987: 9, 15 (as *Cicindela*); Gebert 1991: 176, 187; Wiesner 1992: 175; Werner 2000: 147; Abdel-Dayem et al. 2003: 199; Puchkov and Matalin 2003: 103; Rittner 2003; Abdel-Dayem 2004: 72.

########## Distribution

**Figure 5.**
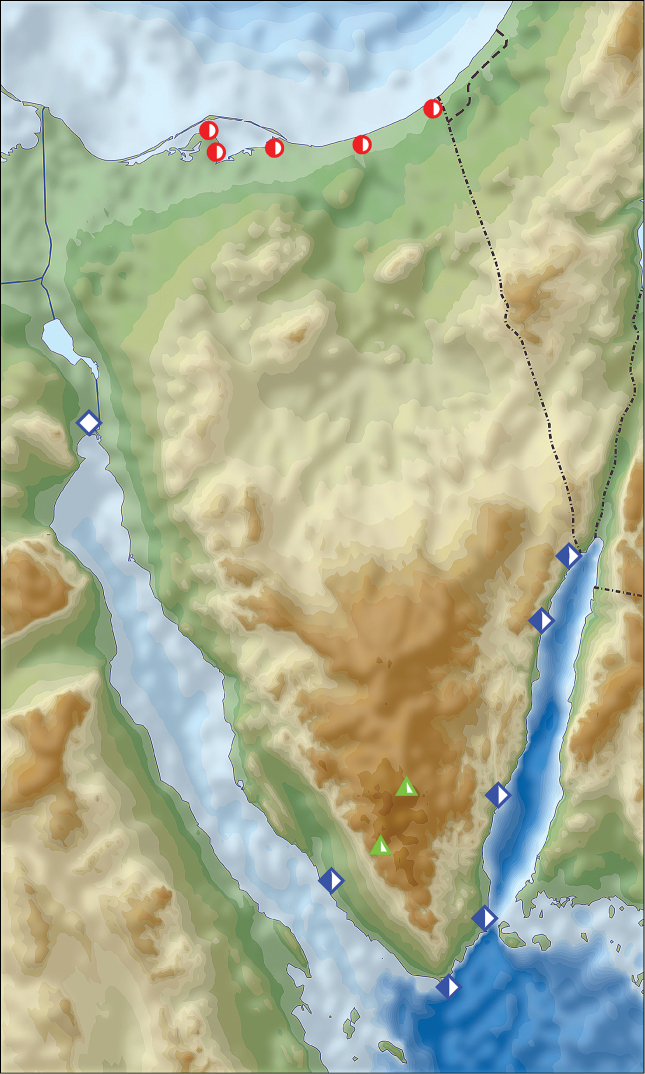
Distribution of *Cephalota tibialis tibialis* (red circles), *Cephalota littorea littorea* (blue rhombs) and *Habrodera nilotica nilotica* (green triangles) in Sinai Peninsula, Egypt (open symbols records before year 1949, half-solid symbols – records between years 1950–1999; URL map source https://upload.wikimedia.org/wikipedia/commons/5/59/Sinai_relief_location_map.svg).

(Fig. 5). **Egypt (Sinai), Sinai Mountains**: *Dahab*, 9.V.1980, leg. A. Valdenberg 1♂ 1♀; *Ras-Burka*, 5.IX.1976, leg. A. Freidberg 1♂ (all TAU); *Sun Pool* (after Nussbaum 1987); **Southwestern Sinai**: *Suez* (after Forskål 1775; Gebert 1991); *Nabeq*, 8.V.1980, 17.VIII.1978, 31.V.1980, leg. A. Valdenberg 25♂♂ 24♀♀; 29.V.1981, leg. A. Freidberg 2♂♂ (all TAU); *El Tor* (after Alfieri 1976; Abdel-Dayem et al. 2003; Abdel-Dayem 2004); *Ras Muhammad*, 16.VIII.1978, leg. A. Valdenberg 2♂♂ 1♀; (after Nussbaum 1987; Gebert 1991).

########## Comments.

The specimen of *Cephalota littorea littorea* (Forskål, 1775) with label “*Jerusalem*” from Zoologisches Museum der Humboldt-Universität (Berlin) is mislabelled (see Gebert 1991). All subsequent records of this species from Israel (Wiesner 1992; Puchkov and Matalin 2003) are in error.

In some publications (Abdel-Dayem et al. 2003; Abdel-Dayem 2004) *Cephalota circumdata* (Dejean, 1822) was recorded from the Sinai Peninsula (El Tor). However, the nominotypical subspecies of *Cephalota circumdata* occurs along the Aegean, Marmora, Black and Mediterranean Sea costs in the Greece, Bulgaria, western Turkey, and, probably Rumania (Franzen 1996; Cassola 1999; Gebert 1999); *Cephalota circumdata cappadocica* Franzen, 1996 and *Cephalota circumdata hattusae* Franzen, 1996 live along banks of the salt lakes in the central Turkey (Franzen 1996; Cassola 1999; Gebert 1999); *Cephalota circumdata leonschaeferi* Cassola, 1970 occupies the Mediterranean sea cost in southern France (including Corsica) and north-western Italia (Gebert 1999); while *Cephalota circumdata imperialis* Klug, 1834 records in the Italia (Sardinia and Sicilia), south-eastern Spain (including Balearic Islands), Tunisia and Algeria, but not in the Libya and Egypt (Gebert 1999). Most likely, the aberrant specimen of *Cephalota littorea* was incorrectly identified as *Cephalota circumdata*.

######### (○) Cephalota (Taenidia) tibialis tibialis

Taxon classificationAnimaliaColeopteraCarabidae

(Dejean, 1882)

########## General distribution.

**Asia** Egypt (Sinai); **Africa** Egypt.

########## References.

**Egypt (Sinai)** – Valdenberg 1983: 42, 47 (as *Cicindela*); Nussbaum 1987: 7, 12 (as *Cicindela*), 1985: 37 (as *Cicindela*); Gebert 1991: 179, 187; Wiesner 1992: 175; El-Moursy et al. 2001: 66 (as *Cicindela littorea*); Abdel-Dayem et al. 2003: 200; Puchkov and Matalin 2003: 103; Abdel-Dayem 2004: 72, 2012: 198.

########## Distribution

(Fig. 5). **Egypt (Sinai), Northern Sinai**: *Yamit*, 21.VI.1978, 14.VII.1981, leg. A. Valdenberg – 3♀♀ (TAU); *Sabkhat al Bardawil*, 7.VI.1977, 26.VII.1978, 31.VIII.1978, 7.VI.1980, leg. A. Valdenberg 4♂♂ 7♀♀ (TAU); *Arish* (after Abdel-Dayem et al. 2003); *Zaranik Protectorate* (after El-Moursy et al. 2001; Abdel-Dayem 2004 2012); *Sabkhat al Shic* (after Nussbaum 1987; Gebert 1991).

######## Genus *Cicindela* Linnaeus, 1758

######### Cicindela (s. str.) javeti azari

Taxon classificationAnimaliaColeopteraCarabidae

Deuve, 2011

########## General distribution.

**Asia** Lebanon, Israel, Syria.

########## References.

**Israel** – Valdenberg 1983: 42, 48 (as *Cicindela campestris herbacea*), 1985: 37 (as *Cicindela campestris herbacea*); Nussbaum 1987: 7-8 (as *Cicindela herbacea*); Wiesner 1992: 127 (as *Cicindela herbacea*); Puchkov and Matalin 2003: 105 (as *Cicindela herbacea*); Rittner 2003 (as *Lophyridia herbacea*); Chikatunov et al. 2006: 293 (as *Cicindela herbacea*); Franzen 2007: 13 (as *Cicindela herbacea*); Ptashkovsky 2009: 8-9 (as *Lophyra herbacea*).

########## Distribution

(Fig. 4). **Israel, Upper Galilee**: *Mt. Meron*, 8.IV.1972, leg. D. Gerling 1♀ (TAU); **Golan Heights**: *Mezudat Nimrod*, 8.V.1983, leg. E. Shney-Dor 3♂♂ 1♀ (TAU); **Mt. Hermon**: 1900 m, 22.IV.1973, leg. D. Furth 1♂; 2000 m, 9.VI.1992, leg. A. Freidberg 1♂ 1♀ (all TAU).

**Lebanon, Liban-Sud**: *Jezzin* 5♂♂ 11♀♀ (after Deuve 2011).

**Syria, Dimashq**: *Bloudan* (after Avgin and Wiesner 2009 as *Cicindela thughurica* Franzen, 2007).

######### (○) Cicindela (s. str.) herbacea herbacea

Taxon classificationAnimaliaColeopteraCarabidae

Klug, 1832

########## General distribution.

**Asia** Lebanon.

########## References.

**Lebanon** – Wiesner 1992: 127; Puchkov and Matalin 2003: 105; Franzen 2007: 13; Deuve 2011: 129.

########## Distribution.

**Lebanon, Liban-Nord**: *Bcharré*, Les Cèdres, VI. 1997 1♂1♀ (cIOv); *Bcharré* 1♀ (after Franzen 2007); *Tannourine* 1♀ (after Deuve 2011).

########## Comments.

Until recently both these species were recorded from Syria, Lebanon and Israel by several authors as *Cicindela herbacea* Klug (Valdenberg 1983; Nussbaum 1987; Wiesner 1992; Puchkov and Matalin 2003; Chikatunov et al. 2006; Franzen 2007; Ptashkovsky 2009). However, according to recent data *Cicindela herbacea* does not occur in Israel (Deuve 2011, 2012). The nominative subspecies occurs in Lebanon and Syria; *Cicindela herbacea aleppensis* Deuve, 2012 is recorded from north-western Syria, while *Cicindela herbacea perreaui* Deuve, 1987 and *Cicindela herbacea colasi* Deuve, 2011 are found in Turkey Tunceli and Adana Provinces, respectively. On the basis of the shape of pronotum (Figs 38 *vs* 39), white elytral pattern (Figs 54 *vs* 55), size of aedeagus and shape of it apex (Figs 93 *vs* 97), as well as shape of internal sack (Figs 94–96 *vs* 98–100) we consider all studied specimens from Israel to be *Cicindela javeti azari* Deuve, 2011 (type locality – Lebanon, Jezzine). It should be noted that the taxonomy of intraspecific forms within the ‘*campestris*’-group is complex, and additional studies are necessary.

######## Genus *Cylindera* Westwood, 1831

######### Cylindera (Eugrapha) contorta valdenbergi

Taxon classificationAnimaliaColeopteraCarabidae

(Mandl, 1981)

########## General distribution.

**Asia** – Israel, Egypt.

########## References.

**Israel** – Bodenheimer 1937: 108 (as *Cicindela*); Mandl 1981: 169 (as *Cicindela*); Naviaux 1983: 79; Valdenberg 1983: 43, 48 (as *Cicindela*), 1985: 29-30 (as *Cicindela*); Nussbaum 1987: 7, 10 (as *Cicindela*); Werner 1992: 22, 48, 74; Wiesner 1992: 195 (as *Cicindina*); Puchkov and Matalin 2003: 110; Rittner 2003 (as *Lophyridia*); Ptashkovsky 2009: 8-9 (as *Lophyridia*).

########## Distribution

(Fig. 4). **Israel, Northern Coastal Plain**: ‘*Akko*, 7.VIII.1980, leg. A. Valdenberg 3♀♀; ‘*Atlit*, 5.VIII.1942, B. Feldman 1♂; *Ma’agan Mikha`el*, 13.VII.1977, 9.IX.1978, 2.V.1979, 26.III.1980, VI.1980, leg. A. Valdenberg 27♂♂ 53♀♀; 27.VII.1979, leg. J. Kugler 2♂♂; 3.VI.1983, leg. E. Shney-Dor 2♂♂ 7♀♀ (all TAU); VII.1987, leg. Y. Nussbaum – 1♀ (SIZ); 17.V.1980, leg. R. Naviaux 1♀; 16.V.1986, leg. Y. Nussbaum 5♂♂ 6♀♀ (both cJW); *Emeq Zevulun* (after Nussbaum 1987). **Central Coastal Plain**: *Bat Yam*, 13.VII.1945, leg. H. Bytinski-Salz 1♂ 4♀♀ (TAU); *Qesariya*, *Zerufa* [*Tsrufa*] (both after Nussbaum 1987).

######## Genus *Habrodera* Motschulsky, 1862

######### (○) Habrodera nilotica nilotica

Taxon classificationAnimaliaColeopteraCarabidae

(Dejean, 1825)

########## General distribution.

**Asia** Egypt (Sinai); **Africa** Canary Islands (Grand Canary), Senegal, Ghana, Mali, Guinea, Equatorial Guinea, Sierra Leone, Nigeria, Central African Republic, Togo, Benin, Sudan, Egypt, Kenya, Congo, Zaire, Tanzania, Ethiopia, Malawi, Mozambique, South Africa.

########## References.

**Israel** – Chikatunov et al. 2006: 293; **Egypt (Sinai)** Alfieri 1976: 2 (as *Cicindela*); Wiesner 1992: 165); Werner 2000: 138; Abdel-Dayem et al. 2003: 202; Puchkov and Matalin 2003: 103; Abdel-Dayem 2004: 74.

########## Distribution

(Fig. 5). **Egypt (Sinai), Sinai Mountains**: *Wadi Isla* (after Alfieri 1976; Abdel-Dayem et al. 2003; Abdel-Dayem 2004); *St. Katherine* (after Abdel-Dayem 2004).

########## Comments.

Previously *Habrodera nilotica nilotica* (Dejean, 1825) was mistakenly referenced in the fauna of Israel (Chikatunov et al. 2006).

######## Genus *Hypaetha* LeCoute, 1857

######### (○) Hypaetha singularis

Taxon classificationAnimaliaColeopteraCarabidae

(Chaudoir, 1876)

########## General distribution.

**Asia** Egypt (Sinai), Oman, Yemen; **Africa** Egypt, Sudan, Somalia, Eritrea, Djibouti.

########## References.

**Egypt (Sinai)** Valdenberg 1983: 43, 45 (as *Cicindela*), 1985: 37 (as *Cicindela*); Nussbaum 1987: 11, 13 (as *Cicindela*); Wiesner 1992: 219; Puchkov and Matalin 2003: 112.

########## Distribution

(Fig. 9). **Egypt (Sinai), Southwestern Sinai**: *Nabeq*, 8.V.1980, leg. A. Valdenberg 1♂; *Ras Muhammad*, 16.VIII.1978, leg. A. Valdenberg 2♂♂ 2♀♀ (all TAU).

######## Genus *Lophyra* Motschulsky, 1859

######### Lophyra (s. str.) flexuosa flexuosa

Taxon classificationAnimaliaColeopteraCarabidae

(Fabricius, 1787)

########## General distribution.

**Europe** Portugal, Spain, Andorra, France, Italy, Switzerland; **Asia** Israel, Egypt (Sinai); **Africa** Morocco, Tunisia, Algeria, Libya, Egypt.

########## References.

**Israel** – Bodenheimer 1937: 108 (as *Cicindela*), Valdenberg 1983: 42, 48 (as *Cicindela flexosa*), 1985: 33 (as *Cicindela flexosa*); Nussbaum 1987: 9, 15 (as *Cicindela*); Wiesner 1992: 160; Puchkov and Matalin 2003: 112; Chikatunov et al. 2006: 293; Ptashkovsky 2009: 8-9; **Egypt (Sinai)** Alfieri 1976: 1-2 (as *Cicindela*); Nussbaum 1987: 9, 15 (as *Cicindela*); Wiesner 1992: 160; Abdel-Dayem et al. 2003: 203; Puchkov and Matalin 2003: 103; Abdel-Dayem 2004: 74.

########## Distribution

**Figure 6.**
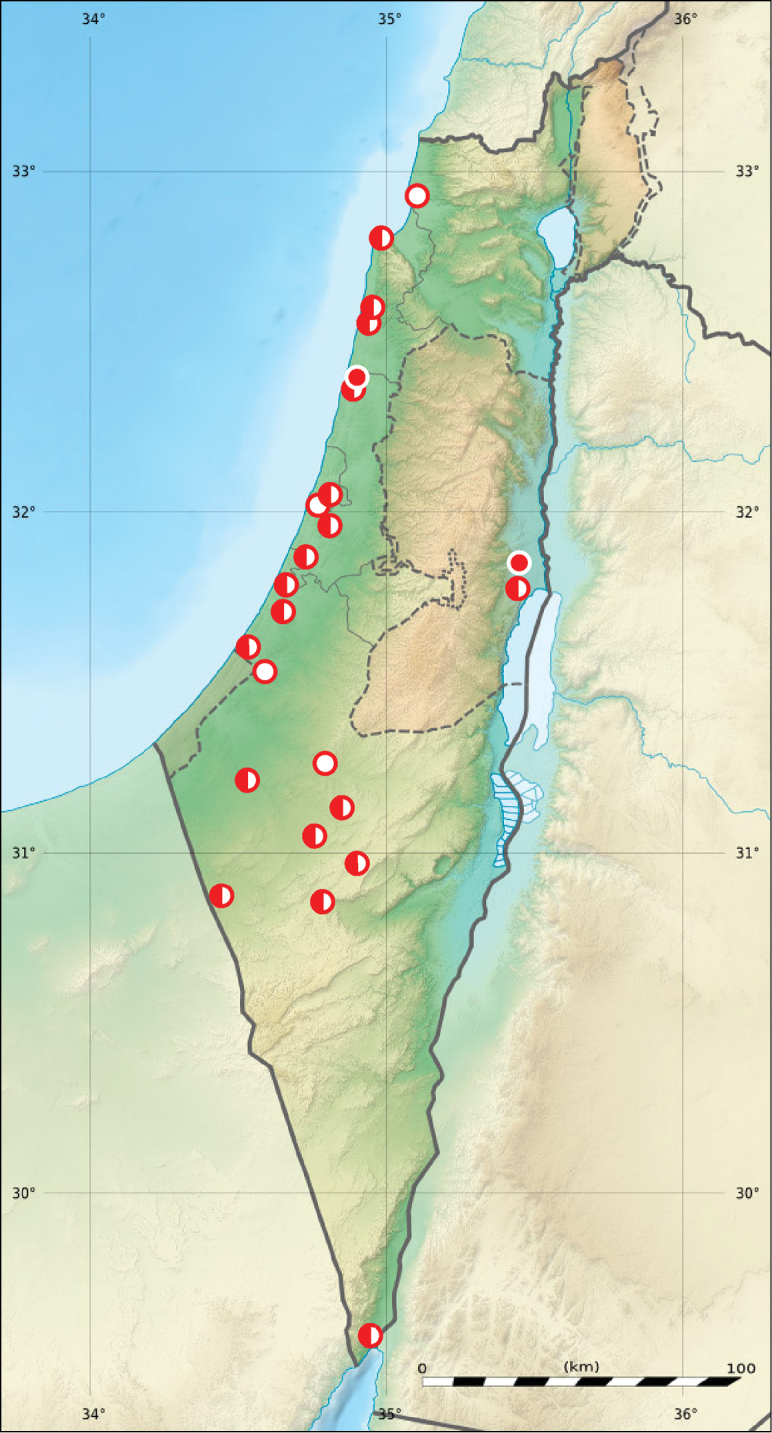
Distribution of *Lophyra flexuosa flexuosa* in Israel and Palestine (open circles records before year 1949, half-solid circles records between years 1950–1999, solid circles records after year 2000; map source Eric Gaba Wikimedia Commons user: Sting and Wikimedia Commons user: NordNordWest, URL https://upload.wikimedia.org/wikipedia/commons/7/7c/Israel_relief_location_map.jpg).

(Figs 6, 9). **Israel (including State of Palestine), Northern Coastal Plain**: ‘*Akko*, 23.IV.1927, leg. O. Theodor 1♀; *Dor*, 23.IV.1998, leg. A. Traub 2♀♀; *Haifa*, 18.V.1996, leg. Hauser 1♀; *Ma’agan Mikha`el*, 16.V.1978, 21.XI.1978, 18.XII.1978, 12.II.1979, 4.III.1979, 10.III.1979, 16.VI.1981, leg. A. Valdenberg 31♂♂ 56♀♀; 16.IV.1983, leg. E. Shney-Dor – 1♂ 3♀♀ (all TAU); **Central Coastal Plain**: *Bat Yam*, 14.III.1940, 12.II.1941, leg. H. Bytinski-Salz 2♂♂ 1♀; *Hofit*, 21.IX.1994, leg. A. Freidberg 1♂; *Holon*, 4.V.1978, leg. A. Freidberg 3♂♂ 2♀♀; *Nahal Alexander*, 32°24'N, 34°52'E, 15.V.2005, leg. I. Zonstein 1♀; *Rehovot*, 18.III.1954, leg. J. Wahrman 2♀♀ (all TAU); **Southern Coastal Plain**: *Ashdod*, sands, 29.II.1984, leg. A. Freidberg 1♂; *Nir ‘Am*, 21.III.1946, leg. H. Bytinski-Salz 2♂♂ 3♀♀; *Nizzanim*, 5.III.1996, leg. A. Freidberg 1♀; *Yavne*, 17.IV.1974, leg. D. Furth 2♂♂; *Ziqqim*, 7.II.1996, leg. I. Yarom & A. Freidberg 2♂♂ (all TAU); **Dead Sea Area**: *Qumeran*, 18.II.1997, leg. V. Chikatunov 2♂♂; *Yeriho*, 23.VII.2002, leg. V. Kravchenko & V. Chikatunov 1♂ 2♀♀ (all TAU); **Arava Valley**: ‘*En ‘Iddan*, 18.IV.1999, leg. I. Yarom & V. Kravchenko 2♂♂ 1♀ (TAU); **Northern Negev**: *Be`er Sheva*, 1.IV.1946, leg. H. Bytinski-Salz 2♀♀; *Bor Mashash*, 18.IV.1995, leg. A. Freidberg 1♂ 1♀; *Gevulot*, 6.IV.1985, leg. E. Shney-Dor 3♂♂ 5♀♀; *Revivim*, Park Golda, 26.III.1999, leg. A. Freidberg 1♂ 1♀ (all TAU); **Central Negev**: *Yeroham*, 27.III.1966, leg. H. Bytinski-Salz 4♂♂ 7♀♀; 25.IV.1973, 22.IV.1981, leg. J. Kugler 1♂ 4♀♀, 19.III.1978, leg. M. Kaplan 6♂♂ 5♀♀; 19.III.1978, leg. A. Freidberg - 6♂♂; *Sede Boker*, 8.VI.1987, leg. E. Shney-Dor – 1♂; *Kadesh Barnea*, 11.IV.1974, leg. D. Furth 3♂♂ 2♀♀; 9.V.1979, leg. A. Valdenberg 3♂♂ 6♀♀ (all TAU); **Southern Negev**: *Elat*, 14.VI.1981, leg. R. Keian 1♀ (TAU).

**Egypt (Sinai), Northern Sinai**: *Sabkhat al Shic*, 8.V.1981, leg. A. Valdenberg 2♂♂ 2♀♀ (TAU); *Ismailia* (after Alfieri 1976); **Central Sinai Foothills**: *Wadi Godirate* [*Gudeirat*] (after Nussbaum 1987: Fig. 6; Abdel-Dayem 2004); **Southwestern Sinai**: *Nabeq* (after Abdel-Dayem 2004), *Wadi Gharandal* (after Alfieri 1976; Abdel-Dayem et al. 2003).

######## Genus *Myriochila* Motschulsky, 1858

######### Myriochila (s. str.) melancholica melancholica

Taxon classificationAnimaliaColeopteraCarabidae

(Fabricius, 1798)

########## General distribution.

**Europe** Portugal, Spain, France, Italy, Malta, Albania, Greece, Georgia, Armenia, Azerbaijan; **Asia** Cyprus, Turkey, Lebanon, Israel, Egypt (Sinai), Syria, Jordan, Saudi Arabia, Arab Emirates, Oman, Yemen, Bahrain, Kuwait, Iran, Iraq, Kazakhstan, Kyrgyzstan, Tadzhikistan, Uzbekistan, Turkmenistan, Afghanistan, Pakistan (Punjab, Sind), India (Punjab, Haryana, Uttar Pradesh, Rajasthan, Madhya Pradesh, Maharashtra, Bihar, West Bengal), China (Xinjiang); **Africa** Cape Verde Islands, Senegal, Morocco, Tunisia, Algeria, Libya, Egypt, Sierra Leone, Guinea, Guinea Bissau, Chad, Ivory Coast, Togo, Ghana, Nigeria, Cameroun, Equatorial Guinea, Central African Republic, Congo, Zaire, Kenya, Somalia, Ethiopia, Sudan, Tanzania, Malawi, Mozambique, Angola, Namibia, South Africa, Madagascar, Seychelles.

########## References.

**Israel** Bodenheimer 1937: 108 (as *Cicindela*); Valdenberg 1983: 43, 46 (as *Cicindela*), 1985: 40 (as *Cicindela*); Nussbaum 1987: 9, 14 (as *Cicindela*); Wiesner 1992: 211; Finkel et al. 2002: 28; Puchkov and Matalin 2003: 114; Rittner 2003; Chikatunov et al. 2006: 293; Ptashkovsky 2009: 8-9; **Egypt (Sinai)** Nussbaum 1987: 9, 14 (as *Cicindela*); Abdel-Dayem et al. 2003: 208; Abdel-Dayem 2004: 75.

########## Distribution

**Figure 7.**
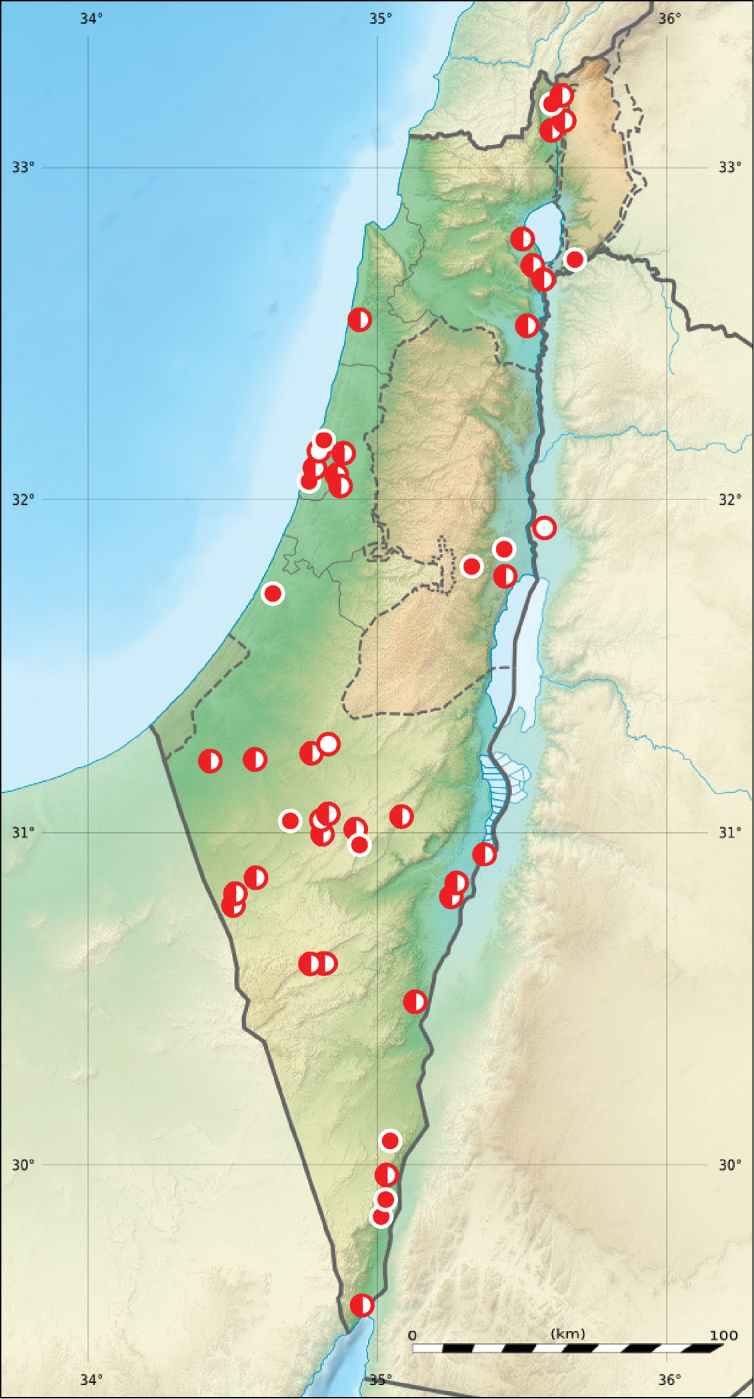
Distribution of *Myriochila melancholica melancholica* in Israel, Palestine and border areas of Jordan (open circles records before year 1949, half-solid circles records between years 1950–1999, solid circles records after year 2000; map source Eric Gaba Wikimedia Commons user: Sting and Wikimedia Commons user: NordNordWest, URL https://upload.wikimedia.org/wikipedia/commons/7/7c/Israel_relief_location_map.jpg).

(Figs 7, 9). **Israel (including State of Palestine), Upper Galilee**: *Tel Dan*, 25.VIII.1958, leg. J. Wahrman 1♂; 23.VIII.2002, leg. V. Kravchenko & V. Chikatunov 2♂♂; *Nahal Keziv*, 28.IX.1999, leg. M. Finkel 1♂ 1♀; *Kefar Szold*, 5.V.1998, leg. R. Ortal 1♂; *Hula*, 1.VI.1968, leg. H. Bytinski-Salz 6♂♂ 3♀♀ (all TAU); **Lower Galilee**: *Teverya*, 3.VI.1961, leg. J. Wahrman 4♂♂ 3♀♀; 24.V.1981, leg. A. Valdenberg 3♂♂ 1♀; *Deganya*, 15.IX.1951, J. Wahrman - 3♂♂ (all TAU); **Golan Heights**: *Hammat Gader*, 23.VII.2002, 2.X.2002, leg. V. Kravchenko & V. Chikatunov 5♂♂ 4♀♀ (TAU); **Northern Coastal Plain**: *Ma’agan Mikha`el*, 17.VI.1973, leg. D. Furth 5♂♂ 7♀♀; 20.IV.1986, leg. A. Freidberg 2♂♂ 3♀♀ (all TAU); **Central Coastal Plain**: *Herzliyya*, 20.V.2000, A. Freidberg 2♀♀; *Bet Dagan*, 26.VIII.1981, leg. Q. Argaman 2♂♂ 3♀♀; *Ramat Gan*, 3.VI.1985, leg. D. Gerling 7♂♂ 5♀♀; *Rosh Ha’Ayin*, 15.X.1994, leg. V. Chikatunov 3♂♂ 4♀♀; *Tel Aviv*, 2.IX.1974, leg. A. Freidberg & M. Kaplan 57♂♂ 60♀♀; 15.VIII.2002, 12.IV.2003, leg. V. Kravchenko & V. Chikatunov 2♂♂ 7♀♀; 24.VII.1948, H. Bytinski-Salz 1♀ (all TAU); **Southern Coastal Plain**: *Nizzanim*, 23.VIII.2002, 5.X.2002, leg. V. Kravchenko & V. Chikatunov 4♂♂ 4♀♀ (TAU); **Judean Desert**: *Nahal Perat (Wadi Qelt)*, 23.VII.2002, leg. V. Kravchenko & V. Chikatunov 1♂ 2♀♀ (TAU); **Jordan Valley**: *Afiqim*, 26.VIII.1971, leg. M. Kaplan 2♂♂; *Ma’oz Hayyim*, 21.V.1977, leg. A. Valdenberg 4♂♂ 2♀♀ (all TAU), from *Dan* to *Ne`ot HaKikkar* (after Nussbaum 1987); **Dead Sea Area**: *Yeriho*, 23.VII.2002, 5.X.2002, leg. V. Kravchenko & V. Chikatunov 3♂♂ 4♀♀; *Qalya*, 6.V.1980, leg. A. Valdenberg 3♂♂ 5♀♀ (all TAU); **Arava Valley**: *Gerofit*, 2.VIII.2002 and 5.X.2002, leg. V. Kravchenko & V. Chikatunov 2♂♂ 4♀♀; *Hazeva*, 19.VII.1999, leg. I. Yarom & V. Kravchenko 2♂♂ 3♀♀; 19.IX.1995, leg. A. Freidberg 1♂ 1♀; ‘*En ‘Iddan*, 20.VI.1995, leg. A. Freidberg 3♂♂ 1♀; *Yotvata*, 24.VIII.1989, leg. A. Eitam 1♂; *Zuqim*, 22.VI.1999, leg. I. Yarom & V. Kravchenko 1♂ 2♀♀; *Samar*, 29°50'N, 35°02'E, 26.IV.2007, leg. N. Ketner 2♂♂ 2♀♀ (all TAU); **Northern Negev**: *Be`er Sheva*, 1.VIII.1945, leg. H, Bytinski-Salz 2♂♂ 2♀♀; *Dimona*, 18.VIII.1957, leg. J. Wahrman 1♂ 1♀; *Gevulot*, 18.V.1983, 6.VI.1984, 29.VIII.1987, leg. E. Shney-Dor 11♂♂ 12♀♀; *Hazerim*, 31.VIII.1951, leg. J. Wahrman 1♂ 1♀; *Retamim*, 12.VI.2002, 5.VI.2003, leg. V. Kravchenko & V. Chikatunov 2♂♂ 5♀♀; *Revivim*, 1.IV.1942, leg. H, Bytinski-Salz 2♂♂ 2♀♀; 2.VIII.1958, leg. J. Kugler 1♂ (all TAU); *Ze`elim*, 17.IX.1986, leg. Y. Nussbaum, 1♀ (cJW); **Central Negev**: *Mash’abbe Sade*, 23.VIII.1965. J. Wahrman - 3♂♂3♀♀; 27.VIII.1986, leg. A. Freidberg 1♂ 1♀; *Yeroham*, 30.V.1957, leg. I. Yarkoni 3♂♂ 1♀; *Tel Yeroham*, 19.XI.1959, leg. L. Fishelsohn 1♂; *Ma`agar Yeroham*, 29.VII.2007, leg. L. Friedman 1♂; 30°59.37'N, 34°53.87'E, 22.V.2008, leg. L. Friedman 2♂♂ 2♀♀; *Makhtesh Ramon*, 9.VIII.1977, leg. A. Freidberg 1♂; *Mizpe Ramon*, 4.VIII.1977, leg. D. Simon 1♂; *Shivta*, 23.VI.1978, leg. A. Freidberg 5♂♂6♀♀ (all TAU); *Qziot*, 8.IX.1986, leg. Y. Nussbaum 2♂♂ (cJW); *Ezuz* (after Nussbaum 1987); **Southern Negev**: *Elat*, 6.IX.1974, leg. A. Freidberg 2♂♂; *Shizzafon*, 12.VI.2002, 5.X.2001, leg. V. Kravchenko & V. Chikatunov 1♂ 2♀♀ (all TAU).

**Jordan, Al Balqā**’: *Al Maghtas*, 23.V.1942, H. Bytinski-Salz 1♀ (TAU).

**Egypt (Sinai), Northern Sinai**: *El Arish*, 15.VI.1968, leg. J. Kugler 1♂ 2♀♀ (TAU; including after Abdel-Dayem et al. 2003; Abdel-Dayem 2004); **Southwestern Sinai**: *Ofira*, sewage, 2.V.1981, leg. A. Freidberg 1♂ (TAU).

###### Tribe Megacephalini Laporte, 1834

####### Subtribe Megacephalina Laporte, 1834

######## Genus *Grammognatha* Motschulsky, 1850

######### Grammognatha euphratica euphratica

Taxon classificationAnimaliaColeopteraCarabidae

Dejean in Latreille & Dejean, 1822

########## General distribution.

**Europe** – Spain, Greece (Rhodes, Crete); **Asia** Cyprus, Turkey, Lebanon, Israel, Jordan, Syria, Egypt (Sinai), Saudi Arabia, Arab Emirates, Kuwait, Oman, Yemen, Iran, Iraq, Pakistan; **Africa** Morocco, Tunisia, Algeria, Libya, Egypt, Djibouti.

########## References.

**Israel** – Bodenheimer 1937: 108 (as *Megacephala*); Naviaux 1983: 75 (as *Megacephala*), Valdenberg 1983: 42, 47, 1985: 40 (as *Megacephala*); Nussbaum 1987: 8, 11 (as *Megacephala*); Wiesner 1992: 44 (as *Megacephala euphratica nigra*); Franzen 2001: 89 (as *Megacephala*); Puchkov and Matalin 2003: 118 (as *Megacephala*); Rittner 2003 (as *Megacephala*); Chikatunov et al. 2006: 293 (as *Megacephala*); Ptashkovsky 2009: 8-9 (as *Megacephala*); **Egypt (Sinai)** Schatzmayr 1936: 6 (as *Megacephala*); Alfieri 1976: 1 (as *Megacephala*); Nussbaum 1987: 8, 11 (as *Megacephala*); Wiesner 1992: 44 (as *Megacephala euphratica nigra*); Werner 1999: 68 (as *Megacephala*); El-Moursy et al. 2001: 66 (as *Megacephala*); Franzen 2001: 88 (as *Megacephala*); Abdel-Dayem et al. 2003: 196; Puchkov and Matalin 2003: 118 (as *Megacephala*); Abdel-Dayem 2004: 73.

########## Distribution

**Figure 8.**
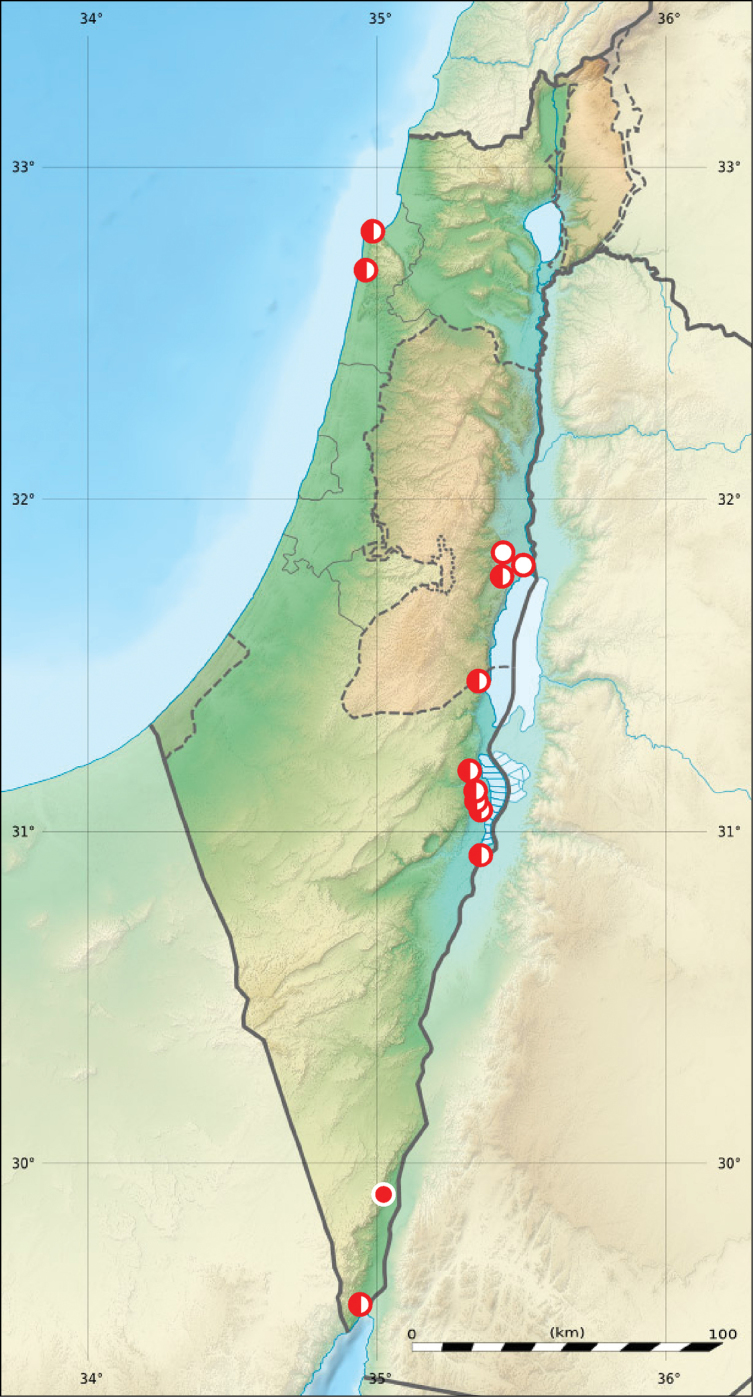
Distribution of *Grammognatha euphratica euphratica* in Israel and Palestine (open circles records before year 1949, half-solid circles records between years 1950–1999, solid circles records after year 2000; map source Eric Gaba Wikimedia Commons user: Sting and Wikimedia Commons user: NordNordWest, URL https://upload.wikimedia.org/wikipedia/commons/7/7c/Israel_relief_location_map.jpg).

**Figure 9.**
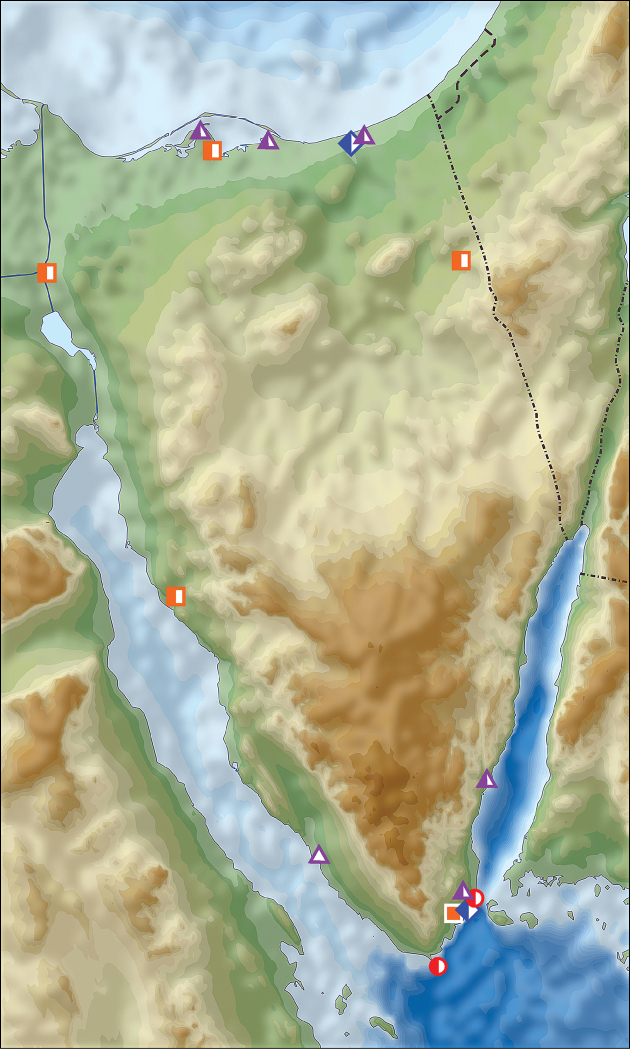
Distribution of *Hypaetha singularis* (red circles), *Myriochila melancholica melancholica* (blue rhombs), *Lophyra flexuosa flexuosa* (orange squares) and *Grammognatha euphratica euphratica* (lilac triangles) in Sinai Peninsula, Egypt (open symbols records before year 1949, half-solid symbols – records between years 1950–1999; URL map source https://upload.wikimedia.org/wikipedia/commons/5/59/Sinai_relief_location_map.svg).

(Figs 8, 9). **Israel (including State of Palestine), Northern Coastal Plain**: *Haifa* (after Franzen 2001); ‘*Atlit*, 4.VI.1979, 1.V.1979, leg. A. Valdenberg 2♀♀; 4.VI.1983, leg. E. Shney-Dor 1♂; 32°42'N, 34°56'E, 17.V.1997, leg. E. Orbach 1♂ 1♀ (all TAU), VI.1989, leg. E. Orbach 1♂ (cJW); V.1989, not far from the coastal line, running to light, leg. E. Orbach 2♂♂ (after Werner 1999); **Dead Sea Area**: *Bet Ha’Arava*, 5.IV.1941, leg. O. Theodor 2♂♂ 2♀♀; *Jordan River*, near Dead Sea, 5.IV.1941, leg. O. Teodor 1♀ (TAU); ‘*En Gedi*, 24.III.1958, leg. J. Kugler 2♂♂; 15.III.65, leg. K. Yefenof 1♀; *Ne`ot HaKikkar*, 15.II.1999, 19.IV.1999, leg. I. Yarom & V. Kravchenko 3♂♂ 1♀; *Qalya*, 11.IV.1958, leg. M. Pener 2.♂♂ 1♀; *Sedom*, 6.V.1961, at night, leg. J. Wahrman 1♂; *Shefekh Zohar*, 16.IV.1980, leg. J. Kugler 1♀; *Zomet Zohar*, 17.IV.1997, leg. L. Friedman 1♀ (all TAU); *Nawit Pools* (after Nussbaum 1987); **Arava Valley**: *Gerofit*, 23.IV.2003, 12.V.2003, 6.VI.2003, leg. D. Utshitel & V. Chikatunov 3♂♂ 2♀♀ (TAU); **Southern Negev**: *Elat*, 30.VIII.1959, leg. L. Fishelsohm – 1 ♂ (TAU).

**Egypt (Sinai), Northern Sinai**: *Sabkhat al Bardawil*, 23.III.1969, leg. A. Nitzan 1♂♂ 2♀♀ (TAU); *Arish* (after Abdel-Dayem 2004); *Zaranik Protectorate* (after El-Moursy et al. 2001; Abdel-Dayem 2004); **Sinai Mountains**: *Dahab* (after Nussbaum 1987); **Southwestern Sinai**: *El Tor* (after Schatzmayr 1936; Alfieri 1976; Nussbaum 1987; Abdel-Dayem et al. 2003; Abdel-Dayem 2004); *Nabeq* (after Nussbaum 1987).

## An identification key to the tiger beetles of Israel and adjacent lands

**Table:** 

1(2)	Anterior angles of pronotum projected towards the margin of prothorax (Figs 10, 45); the fourth joint of maxillary palpus shorter than the third one	**Megacephalini** (*Grammognatha euphratica euphratica* Dejean, 1822)
2(1)	Anterior angles of pronotum not projected towards the margin of prothorax (Figs 12–14); the fourth joint of maxillary palpus longer than the third one	**Cicindelini 3**
3(4)	Proepisterna prominent on pronotum so pronotopleural suture clearly visible dorsally (Fig. 42); anterior margin of pronotum with row of flat white setae	***Hypaetha*** ((○) *Hypaetha singularis* (Chaudoir, 1876))
4(3)	Proepisterna not prominent on pronotum so pronotopleural suture not visible dorsally (Figs 30–41, 43–44); anterior margin of pronotum glabrous	**5**
5(6)	Labrum with four submarginal setae (Fig. 29); middle and hind femora with numerous hooked setae along posterior margin, hind femora with sparse hooked setae (Fig. 64)	***Myriochila* (s. str.)** (Myriochila (s. str.) melancholica melancholica (Fabricius, 1798))
6(5)	Labrum with at least six submarginal setae, except aberrant specimens with 3–5 setae (Figs 15–26, 28); femora without hooked setae along posterior margin	**7**
7(8)	Genae pilose (Figs 11, 14)	**9**
8(7)	Genae glabrous (Figs 12–13)	**13**
9(10)	Clypeus glabrous, anterior and posterior margins of each eye with group of white decumbent setae; labrum with 10 submarginal setae in a single row (Fig. 25); fourth antennomere of males with penicillus (Fig. 14); white elytral pattern with complete humeral lunule, long sinuate middle band and apical lunule coupling together via marginal and sutural bands (Fig. 56)	***Habrodera* ((○)** *Habrodera nilotica nilotica* (Dejean, 1825))
10(9)	Clypeus pilose, anterior and posterior margins of each eye glabrous; labrum with several rows of numerous submarginal setae (Figs 15–18); fourth antennomere of males glabrous (Fig. 11); white elytral pattern without marginal and sutural bands (Figs 46–49)	***Calomera* 11**
11(12)	Elytra dark brown with purple-bronze or green reflection (Fig. 46); pronotum 1.05–1.15 times as wide as long with straight parallel or slightly convergent lateral sides (Fig. 30); aedeagus straight, with long thin basal portion, apical lobe with distinct lateral flanges and small hook, without central groove (Figs 73, 77), ventro-apical bladder of internal sac short, right and left basi-lateral bladders very large (Figs 77, 81)	***Calomera aulica aulica* (Dejean, 1831)**
12(11)	Elytra green sometimes with bronze or blue reflection (Figs 47–49); pronotum 1.15–1.35 times as wide as long with rounded distinctly convergent lateral sides (Figs 31–33); aedeagus curved, with short basal portion, apical lobe without lateral flanges and hook, but with clear central groove (Figs 74–76, 82–84), ventro-apical bladder of internal long, right and left basi-lateral bladders as small acicular areas (Figs 78–80, 82–84)	***Calomera littoralis* (Fabricius, 1787) 12a**
12a(12b)	Left mandible with four teeth distal to apical molar (Fig. 16); pronotum narrow, 1.15–1.2 times wider than long (Fig. 31); aedeagus with small distinct bulge on the dorsal surface (Fig. 74); ventro-apical bladder of internal sac long and curved towards and on the left, apex of medial tooth blunt (Figs 74, 78, 82)	***Calomera littoralis aulicoides* (J.R. Sahlberg, 1913)**
12b(12a)	Left mandible with three teeth distal to apical molar (Fig. 17–18); pronotum wide, 1.2–1.35 times wider than long (Figs 32–33); aedeagus without bulge on the dorsal surface (Figs 75–76); ventro-apical bladder of internal sac straight and not curved, apex of medial tooth sharp (Figs 75–76, 79–80, 83–84)	**12c**
12c(12d)	Labrum wider, 2.6–2.65 times as wide as long (Fig. 32), base of medial tooth of internal sac with one rarely two small additional spikes (Figs 75, 79, 83)	***Calomera littoralis winkleri* (Mandl, 1934)**
12d(12c)	Labrum narrower, 2.35–2.45 times as wide as long (Fig. 33), base of medial tooth of internal sac smooth, without additional spikes (Figs 76, 80, 84)	**(○) *Calomera littoralis nemoralis* (Olivier, 1790)**
13(14)	Labrum tridentate with distinctly prominent apical teeth; mandibles with two teeth distal to apical molar (Fig. 26); scapus covered by numerous white decumbent setae (Figs 13, 26), fourth antennomere of males with penicillus (Fig. 13); posterior margin of each eye with group of white decumbent setae; white elytral pattern with basal dot and incomplete sutural band (Fig. 57)	***Lophyra* (s. str.)** (Lophyra (s. str.) flexuosa flexuosa (Fabricius, 1787))
14(13)	Labrum unidentate (Fig. 20–24, 28), in some species tridentate but with not or slightly prominent apical teeth only (Fig. 19); mandibles with three teeth distal to apical molar (Fig. 19–24); scapus glabrous (Figs 19–22, 28) or only with several sparse setae except apical ones (Figs 23–24), fourth antennomere of males glabrous (Fig. 11); posterior margin of each eye glabrous; white elytral pattern without basal dots and sutural band (Figs 50–55, 59)	**15**
15(16)	Head glabrous; scapus with apical setae only (Fig. 19–22, 28); lateral side of pronotum pilose (Figs 34–37, 43); white elytral pattern with long marginal band and long sinuate middle band (Figs 50–53, 59)	**17**
16(15)	Frons and vertex with long soft hairs, scapus with several setae except apical ones (Figs 23–24); lateral side of pronotum with soft sparse setae (Figs 38–39); white elytral pattern without marginal band and only with short slightly curved middle band (Figs 54–55)	***Cicindela* (s. str.) 25**
17(18)	Anterior portion of apical lunule long, extending basal transverse portion of middle band (Fig. 59)	**Cylindera (Eugrahpa)** (Cylindera (Eugrapha) contorta valdenbergi (Mandl, 1981))
18(17)	Anterior portion of apical lunule short, extending only apical portion of middle band (Figs 50–53)	**Cephalota (Taenidia) 19**
19(20)	Labrum tridentate, relatively short, no less than 2.3 times as wide as long (Fig. 19); pronotum 1.2–1.4 times wider than long (Fig. 34); mesepisternum entirely covered by white setae, densely in males and sparsely in females; white elytral pattern with relatively broad marginal band coupling with humeral and apical lunule as well as with middle band (Fig. 50), apical margin of elytra in sexes wide rounded, subtend practically right angle with sutural tooth (Figs 65–66); aedeagus with long thin gradually curved basal portion (Fig. 85)	**(○) Cephalota (Taenidia) tibialis tibialis (Dejean, 1822)**
20(19)	Labrum unidentate, relatively long, no more than 2.3 times as wide as long (Figs 20–22); pronotum 1.1–1.25 times wider than long (Figs 35–37); mesepisternum covered by white setae only along posterior margin and on the base; white elytral pattern usually with narrow marginal band or without it so in some specimens humeral lunule distinctly separated (Figs 51–53), apical margin of elytra at least in males subtend acute angle with sutural tooth (Figs 67–72); aedeagus with short thin basal portion (Figs 87, 89, 91)	**21**
21(22)	4–11^th^ antennomeres dark brown; elytra bright purple, 1.5–1.6 times as long as wide (Fig. 51), apical elytral margin in females narrowly rounded and subtend small right angle with sutural tooth (Fig. 67–68); aedeagus with broad blunt apex (Figs 87–88)	**(○) Cephalota (Taenidia) littorea littorea (Forskål, 1775)**
22(21)	4–11^th^ antennomeres light brown or yellowish; elytra greenish or greenish-blue sometimes with distinct golden-purple reflection, no less than 1.65 times as long as wide (Figs 52–53), apical elytral margin in both sexes subtend acute angle with sutural tooth (Figs 69–72); aedeagus with arrow-shaped apex (Figs 89–92)	**23**
23(24)	Labrum shorter, 2.0–2.2 times as wide as long (Fig. 21); lateral side of pronotum straight, slightly convergent to large posterior angles (Fig. 36); humeral lunule separated or narrowly coupled with marginal band (Fig. 52); aedeagus larger, with relatively long thin basal portion (Fig. 89) and short tapered apex (Fig. 90)	**Cephalota (Taenidia) zarudniana vartianorum (Mandl, 1967)**
24(23)	Labrum longer, 1.6–1.7 times as wide as long (Fig. 22); lateral side of pronotum slightly rounded, distinctly convergent to small posterior angles (Fig. 37); humeral lunule coupled with middle band via marginal band (Fig. 52); aedeagus smaller, with short thin basal portion (Fig. 91) and long tapered apex (Fig. 92)	**(○) Cephalota (Taenidia) deserticola deserticola (Faldermann, 1836)**
25(26)	Pronotum with convex lateral sides gradually convergent to posterior angles, anterior margin same length or slightly longer than posterior one, notopleural suture looks like smooth border (Fig. 39); mesepisternum of female with small shallow pit and deep all along coupling sulcus, mesepimeron with groove along anterior margin (Fig. 63); middle band of white elytral pattern without oblique strip between transverse basal and circled apical portions, basal portion of apical lunule small (Fig. 55); aedeagus shorter, no more than 0.55 times as long as elytra (Fig. 97); basal and right ventro-lateral bladders of internal sac short (Figs 98–100)	**Cicindela (s. str.) javeti azari Deuve, 2011**
26(25)	Pronotum with straight lateral sides sharply convergent to posterior angles, anterior margin clearly longer than posterior one, notopleural suture looks like cut border (Fig. 38); mesepisternum of female with deep apically but shallow and indistinct basally coupling sulcus only, mesepimeron without groove along anterior margin (Fig. 62); middle band of white elytral pattern with distinct oblique strip between transverse basal and circled apical portions, basal portion of apical lunule large (Fig. 54); aedeagus longer, no less than 0.6 times as long as elytra (Fig. 93); basal and right ventro-lateral bladders of internal sac long (Figs 94–96)	**(○) Cicindela (s. str.) herbacea herbacea Klug, 1832**

**Figures 10–14.**
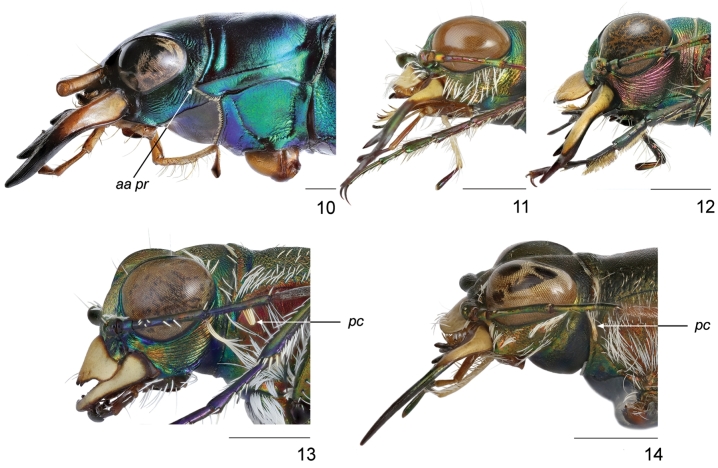
Head and pronotum of males, left lateral view: **10** *Grammognatha euphratica euphratica* **11** *Calomera aulica aulica* **12** *Cicindela javeti azari* **13** *Lophyra flexuosa flexuosa* **14** *Habrodera nilotica*; *aa pr* – anterior angle of pronotum; *pc* – penicillus. Scale bars: 1 mm.

**Figures 15–22.**
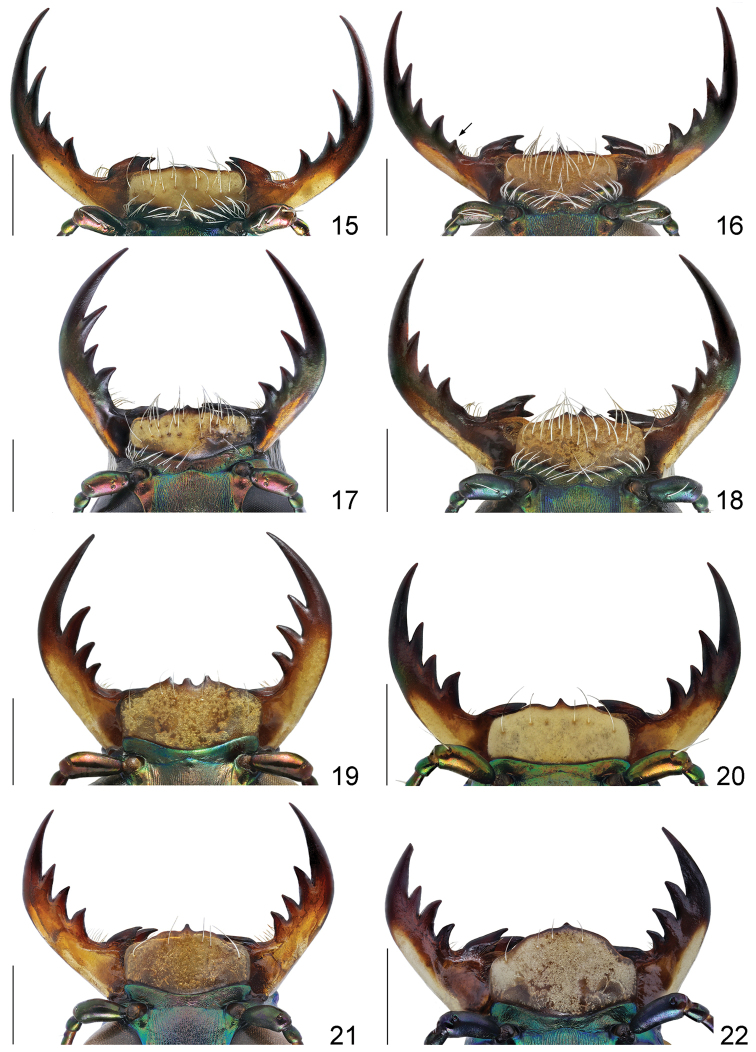
Labrum and mandibles of males, dorsal view: **15** *Calomera aulica aulica* **16** *Calomera littoralis aulicoides* **17** *Calomera littoralis winkleri* **18** *Calomera littoralis nemoralis* **19** *Cephalota tibialis tibialis* **20** *Cephalota littorea littorea* **21** *Cephalota zarudniana vartianorum* **22** *Cephalota deserticola deserticola*. Scale bars: 1 mm.

**Figures 23–29.**
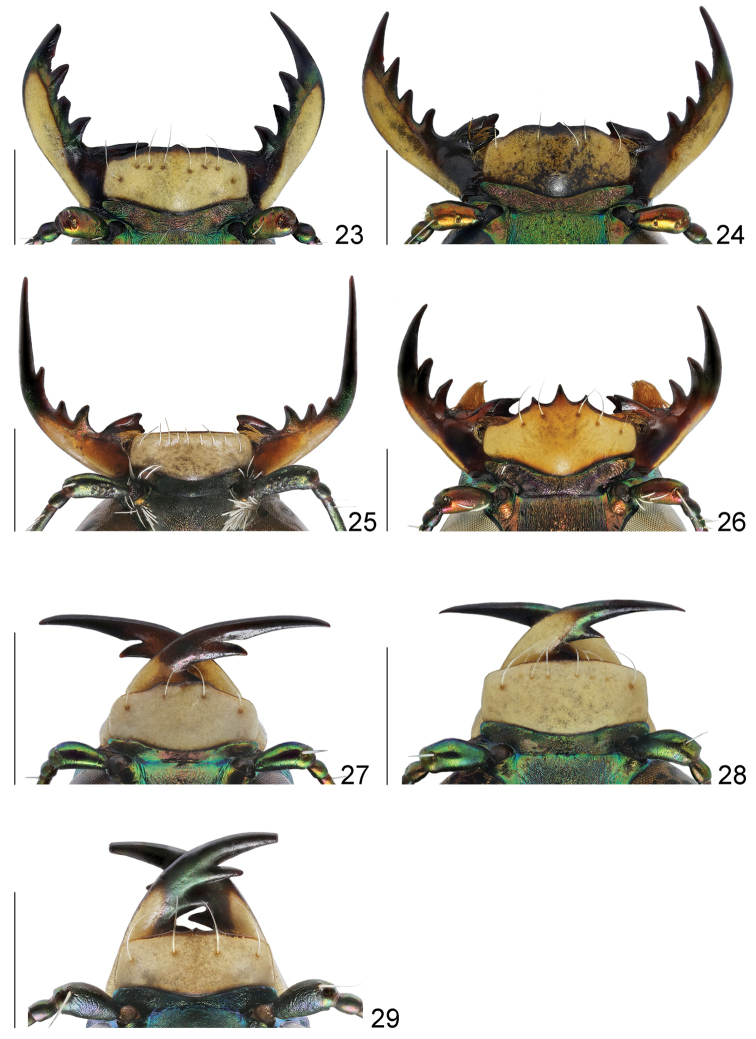
Labrum and mandibles of males, dorsal view: **23** *Cicindela herbacea herbacea* **24** *Cicindela javeti azari* **25** *Habrodera nilotica nilotica* **26** *Lophyra flexuosa flexuosa* **27** *Hypaetha singularis* **28** *Cylindera contorta valdenbergi* **29** *Myriochila melancholica melancholica*. Scale bars: 1 mm.

**Figures 30–45.**
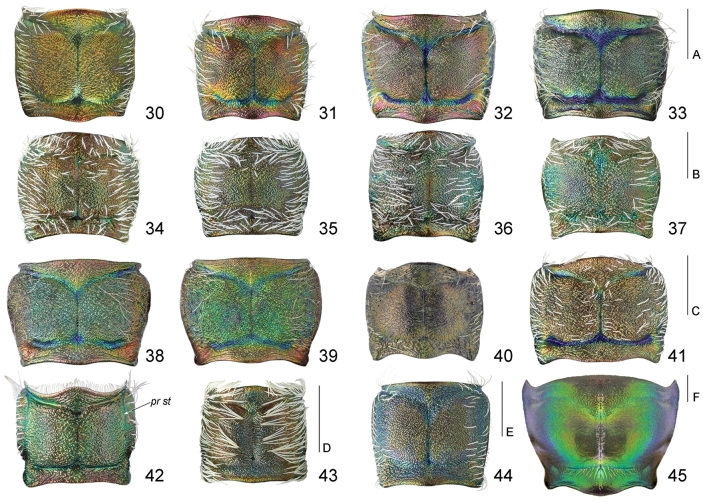
Pronotum of males, dorsal view: **30** *Calomera aulica aulica* **31** *Calomera littoralis aulicoides* **32** *Calomera littoralis winkleri* **33** *Calomera littoralis nemoralis* **34** *Cephalota tibialis tibialis* **35** *Cephalota littorea littorea* **36** *Cephalota zarudniana vartianorum* **37** *Cephalota deserticola deserticola* **38** *Cicindela herbacea herbacea* **39** *Cicindela javeti azari* **40** *Habrodera nilotica nilotica* **41** *Lophyra flexuosa flexuosa* **42** *Hypaetha singularis* **43** *Cylindera contorta valdenbergi* **44** *Myriochila melancholica melancholica* **45** *Grammognatha euphratica euphratica*; *pr st* pronotopleural suture. Scale bars: 1 mm (30–33: A; 34–37: B; 38–41: C; 42–43: D; 44: E; 45: F).

**Figures 46–61.**
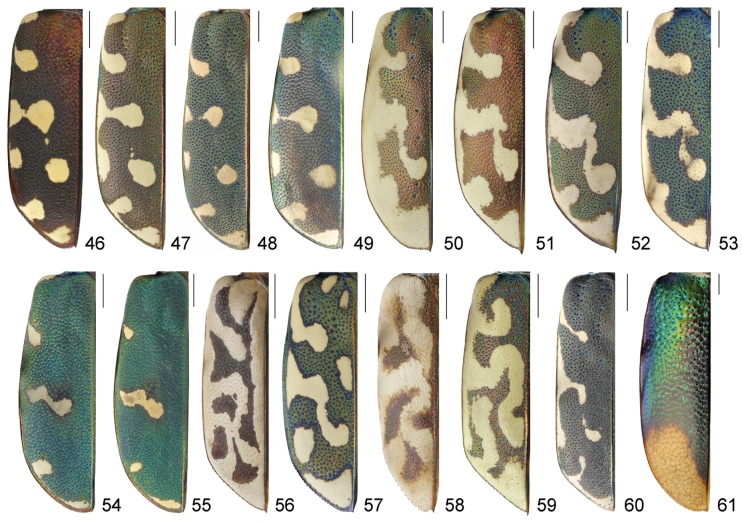
Left elytron of males, dorsal view: **46** *Calomera aulica aulica* **47** *Calomera littoralis aulicoides* **48** *Calomera littoralis winkleri*; **49** *Calomera littoralis nemoralis* **50** *Cephalota tibialis tibialis* **51** *Cephalota littorea littorea* **52** *Cephalota zarudniana vartianorum* **53** *Cephalota deserticola deserticola* **54** *Cicindela herbacea herbacea* **55** *Cicindela javeti azari* **56** *Habrodera nilotica nilotica* **57** *Lophyra flexuosa flexuosa* **58** *Hypaetha singularis* **59** *Cylindera contorta valdenbergi* **60** *Myriochila melancholica melancholica* **61** *Grammognatha euphratica euphratica*. Scale bars: 1 mm.

**Figures 62–72.**
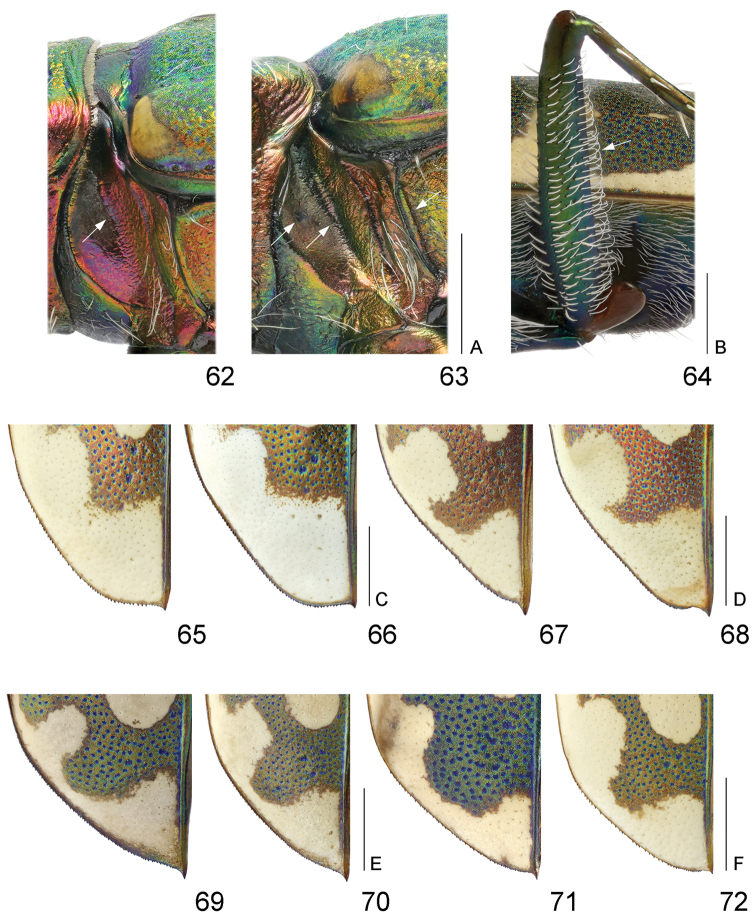
Details of Cicindelinae: **62–63** mesoepisternal coupling sulcus **64** hind femora **65–72** apical part of left elytron **62** *Cicindela herbacea herbacea* **63** *Cicindela javeti azari* **64** *Myriochila melancholica melancholica* **65–66** *Cephalota tibialis tibialis* **67–68** *Cephalota littorea littorea* **69–70** *Cephalota zarudniana vartianorum* **71–72** *Cephalota deserticola deserticola* **64–65, 67, 69, 71** males **62–63, 66, 68, 70, 72** females. Scale bars: 1 mm (62–63: A; 64: B; 65–66: C; 67–68 D; 69–70: E; 71–72: F).

**Figures 73–76.**
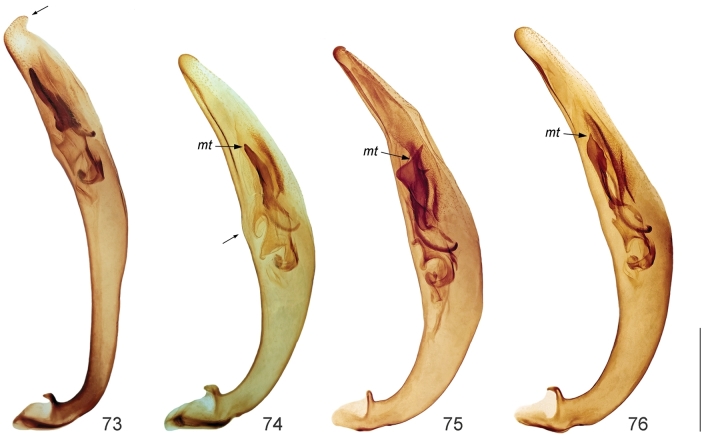
Aedeagus of *Calomera* spp., right lateral view: **73** *Calomera aulica aulica* **74** *Cicindela littoralis aulicoides* **75** *Calomera littoralis winkleri* **76** *Calomera littoralis nemoralis*; *mt* – median tooth. Scale bar: 1 mm.

**Figures 77–84.**
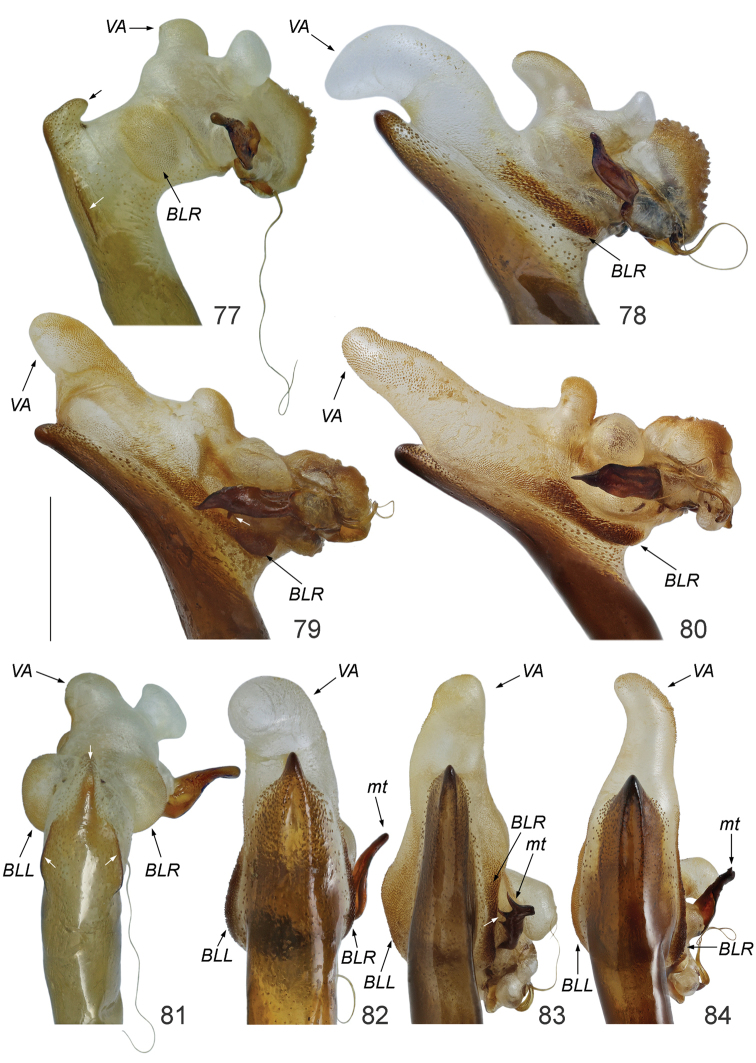
Internal sack of *Calomera* spp.: **77, 81** *Calomera aulica aulica* **78, 82** *Cicindela littoralis aulicoides* **79, 83** *Calomera littoralis winkleri* **80, 84** *Calomera littoralis nemoralis* **77–80** right lateral view **81–84** dorsal view; *BLR* basi-lateral right bladder; *BLL* basi-lateral left bladder; *VA* – ventro-apical bladder; *mt* – median tooth. Scale bar: 1 mm.

**Figures 85–92:**
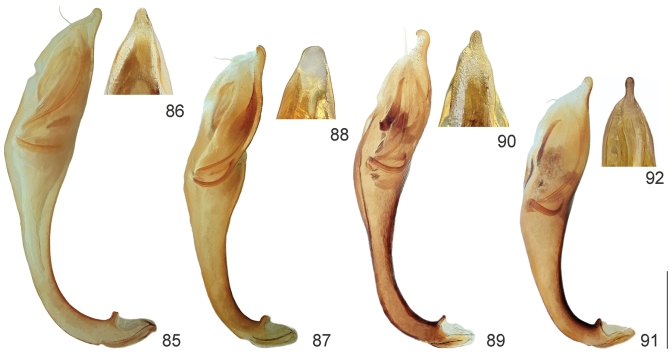
. Aedeagus of *Cephalota* spp.: **85–86** *Cephalota tibialis tibialis* **87–88** *Cephalota littorea littorea* **89–90** *Cephalota zarudniana vartianorum* **91–92** *Cephalota deserticola deserticola* **85, 87, 89, 90** aedeagus, left lateral view **86, 88, 91, 92** apex of aedeagus, ventral view. Scale bar: 1 mm.

**Figures 93–100.**
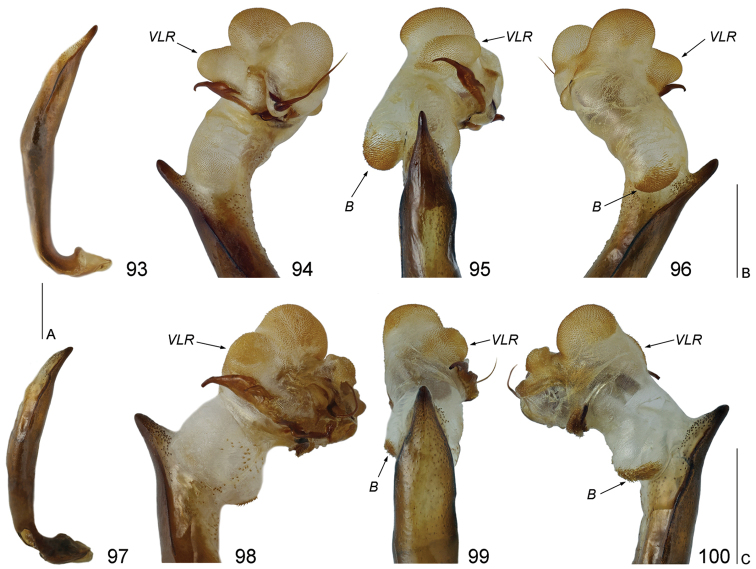
Aedeagus and internal sack of *Cicindela* ssp.: **93–96** *Cicindela herbacea herbacea* **97–100** *Cicindela javeti azari* **93, 97** aedeagus **94–96, 98–100** internal sac **93, 96–97, 100** left lateral view **95, 99** dorsal view **94, 98** right lateral view **98–100** partly inflanted); *B* basal bladder; *VLR* – ventro-lateral right bladder. Scale bars: 1 mm (93, 97: A; 94–96: B; 98–100: C).

**Figure 101.**
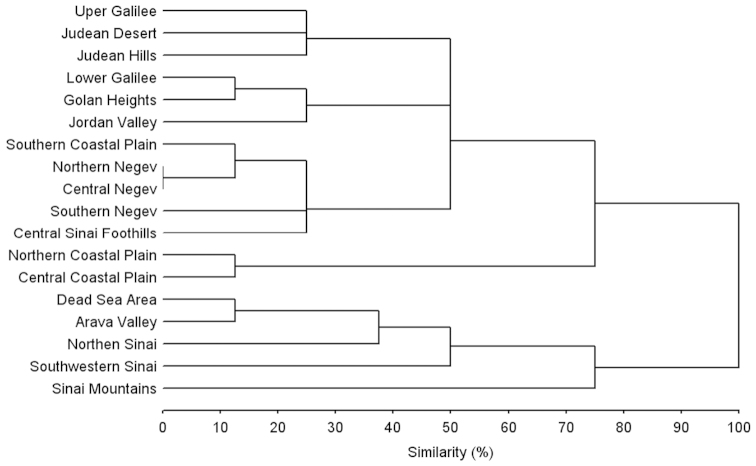
Similarities between tiger beetle faunas of different regions of Israel and the Sinai Peninsula (Complete linkage procedure, squared Euclidean distances).

## Distribution

With these current records, eight species of tiger beetles, one of them with two subspecies, belonging to seven genera of two tribes are known from Israel (Table 1). The Rift Valley, including Jordan Valley, Dead Sea area and Arava Valley, with six cicindelids species is the most speciose region. The Coastal Plain is the second richest region with five species. The species richness gradually decreases from Northern (fife species) through Central (four species) to Southern (three species) Coastal Plain. In the central densely populated areas of Israel, such as Samaria and Judea, the least number of tiger beetles species are recorded. Among all *Myriochila melancholica melancholica* is the most common species observed in all regions of the country (Table 1, Fig. 7), while *Lophyra flexuosa flexuosa* is the second most widespread species of tiger beetles absent only from northern (Galilee, Golan Heights) and central (Samaria, Judea) regions (Table 1, Fig. 6). *Lophyra flexuosa* (Fabricius, 1787) reaches the eastern limit of its distribution in Israel.

**Table 1.** The distribution of tiger beetles in different regions of Israel and the Sinai Peninsula.

Species/Subspecies	Choro-types	Israel														Siani (Egypt)			
		Galilee		Golan Heights (including Mt. Hermon)	Coastal Plain			Judea		Rift Valley			Negev			Northern Siani	Central Siani Foothills	Sinai Mountains	Southwestern Sinai
		Upper	Lower		Northern	Central	Southern	Judean Desert	Judean Hills	Jordan Valley	Dead Sea Area	Arava Valley	Northern	Central	Southern				
*Calomera aulica aulica*	SSS									●	●	●				●		●	●
*Calomera littoralis aulicoides*	NAA		●	●						●	●	●							●
*Calomera littoralis winkleri*	SAT	●			●	●	●		●										
*Cephalota zarudniana vartianorum*	INP										●								
*Cephalota tibialis tibialis*	EGYP															●			
*Cephalota littorea littorea*	NAA																	●	●
*Cicindela javeti azari*	LEVC	●		●															
*Cylindera contorta valdenbergi*	CPND				●	●													
*Habrodera nilotica nilotica*	AFT																	●	
*Hypaetha singularis*	NAA																		●
*Lophyra flexuosa flexuosa*	WMA				●	●	●				●	●	●	●	●	●	●		●
*Myriochila melancholica melancholica*	AMC	●	●	●	●	●	●	●		●	●	●	●	●	●	●			●
*Grammognatha euphratica euphratica*	SMS				●						●	●			●	●		●	●
Total for localities		3	2	3	5	4	3	1	1	3	6	5	2	2	3	5	1	4	7
		4		3	5			2		6			3			9			
Total for regions		8(9)														9			

Chorotypes: AFT – Afrotropical , AMC – Afrotropical-Mediterraneo-Centralasiatic , INP – Irano-Palestinian , NAA – NE-African-Arabian , SAT – S-Anatolia-Turanian , SMS – S-Mediterraneo-Sindian , SSS – Saharo-Sahelo-Sindian , WMA – W-Mediterraneo-N-African , CPND – Coastal Plain-Nile Delta endemic , EGYP – Egyptian endemic , LEVC – C-Levntian endemic .

Three subspecies, *Cylindera contorta valdenbergi*, *Cicindela javeti azari* and *Cephalota zarudniana vartianorum*, are characterized by a restricted distribution in Israel (Fig. 4). The first two first subspecies should be considered as regional endemics.

The nominative subspecies of *Cylindera contorta* (F.-W., 1828) is widely distributed in Central Asia, some regions of Cis- and Transcaucasia as well as in the northern and western sides of the Black Sea from southern Russia to Romania (Wiesner 1992; Cassola 1999; Puchkov and Matalin 2003), however it is not known from Anatolia (Corel 1988; Cassola 1999; Puchkov and Matalin 2003; Avgin and Özdikmen 2007), Syria (Wiesner 1992; Puchkov and Matalin 2003; Avgin and Wiesner 2009; Jaskuła and Rewicz 2014), Jordan (Wiesner 1992; Puchkov and Matalin 2003), Iraq (Ali 1978; Wiesner 1992; Puchkov and Matalin 2003) and Saudi Arabia (Wiesner 1992; Cassola and Schneider 1997; Puchkov and Matalin 2003; Al Ahmadi and Salem 1999). The populations of *Cylindera contorta valdenbergi* inhabit the Mediterranean coast from ‘Akko (Northern Coastal Plain) to Bat Yam (Central Coastal Plain) in Israel (Nussbaum 1987; our data) as well as between Ras El Bar and Abu Qir in north-eastern Egypt (Alfieri 1976; Abdel-Dayem et al. 2003) are distinctly scattered and bound the southwestern limit of the distributional area of *Cylindera contorta* as a whole.

*Cicindela javeti azari* has a restricted distributional area and now is known only from southern Lebanon (Deuve 2011), southwestern Syria (Avgin and Wiesner 2009) as well as northern regions of Israel: Upper Galilee and Golan Heights (Nussbaum 1987; our data). Among three known subspecies (Deuve 2011) *Cicindela javeti azari* inhabits the southern part of the species range area.

*Cephalota zarudniana vartianorum* lives from south-eastern Iran across Iraq and Syria to Jordan and Israel (Wiesner 1992; Puchkov and Matalin 2003). The Dead Sea Area is the western border of the distributional area both for this subspecies as well as for the species as a whole.

It should be noted that the three mentioned above subspecies were recorded in Israel only during XX century (Fig. 4), and the latest records are dated from the late 80’s to the early 90’s.

The Sinai Peninsula is the most diversity of tiger beetles region from all neighbouring territories by Israel because nine species live here, and *Cephalota tibialis tibialis*, *Cephalota littorea littorea*, *Hypaetha singularis* and *Habrodera nilotica nilotica* are never really observe in Israel (*vs* Chikatunov et al. 2006). Among them *Cephalota tibialis tibialis* is an endemic of Egypt and occurs along Mediterranean Sea coast in the Governorates Matrouh, Alexandria, Kafr el-Sheikh, Damietta, Port Said and North Sinai (Gebert 1991; Abdel-Dayem et al. 2003; Abdel-Dayem 2012). Moreover, *Cephalota littorea littorea* is an regional near-endemic living along Red Sea coast in Egypt and Saudi Arabia (Gebert 1991; Cassola and Schneider 1997; Abdel-Dayem et al. 2003). Arabian-African *Hypaetha singularis* lives along Red Sea coast in Egypt, Sudan, Eritrea and Yemen, and on the shore of Gulf of Aden in Djibouti, Somalia and Yemen (Wranik et al. 1991; Werner 2000; Wiesner 2002, 2005) as well as on the littoral of Arabian Sea in Oman (Cassola and Rihane 1996). The Sinai localities are limited the northern border of the distribution area of this species. African *Habrodera nilotica nilotica* is widely distributed in Afrotropical Region (Wiesner 1992; Werner 2000). Two known localities from Sinai Mountains (Alfieri 1976; Abdel-Dayem et al. 2003; Abdel-Dayem 2004) are limited the distribution range of this species to the east.

According to the analysis of the similarity between faunas of tiger beetle of natural regions of Israel and the Sinai Peninsula two large clusters are recognized (Fig. 101). First of them includes the faunas associated with southern part of the Great Rift Valley (Arava valley and Dead Sea area) and most part of the Sinai Peninsula, while the second combine most Israeli regions as well as Central Sinai Foothills. The last cluster diverges on the four groups. The fist combines assemblages of tiger beetles of the Mediterranean coastal habitats within the Northern and Central Coastal Plains. The communities typical for the arid habitats of the Negev Desert and the Central Sinai Foothills as well as for coastal habitats of the Southern Coastal Plain form the second group. The third group includes assemblages of the northern not seashore habitats of the Jordan Valley, Lower Galilee and Golan Heights. The last group is artificial, because the fauna of tiger beetles of Judea should be most similar to the fauna of the Dead Sea Area or the Northern Negev, while the fauna of tiger beetles of the Galilee, Jordan Valley and Golan Heights should be the most similar to each other. First of all, this discrepancy is due to a lack of data about tiger beetles of the central regions of Israel.

## Phenology

According to the literature data (Alfieri 1975; Nussbaum 1987; Abdel-Dayem et al. 2003) and the results of our own study some aspects of the phenology of tiger beetles both in Israel and on the Sinai Peninsula are discussed. The period of activity of the beetles but not the breeding period was analysed first of all. As a result, five groups of the tiger beetles were obtained (Table 2). Three species with the longer period of activity from January to November or from February to December belong to the all-year group. Five species, including two subspecies of *Calomera littoralis* (F., 1787), characterized by the prolonged period of activity from February to October-November, from March-April to November or from March to December and form the richest spring-fall group. Two species recorded only on the Sinai Peninsula with the period of activity from May to August-September are composed the summer group. At last, both the spring group (activity from February to May) and the spring-summer group (activity from February to August) contain a single species each.

**Table 2.** The phenology of tiger beetles in Israel (grey – our data; pink – after Nussbaum 1987) and in the Egypt (green – after Alfieri 1975; blue – after Abdel-Dayem et al. 2003).

*Cephalota zarudniana vartianorum*													*Spring*
											
*Grammognatha euphratica euphratica*			*(Si)*										*Spring-summer*
											
						*(Si)*							
											
*Hypaetha singularis*					*Si*			*Si*					*Summer*
											
						*Si*	*Si*						
*Cephalota littorea littorea*					*Si*			*Si*	*Si*				
					*Si*	*Si*	*Si*	*Si*	*Si*				
						*(Si)*		*(Si)*					
								*(Si)*					
*Cephalota tibialis tibialis*						*Si*	*Si*	*Si*					*Spring-fall*
					*Si*	*Si*	*Si*	*Si*	*Si*				
		*Si*	*Si*	*Si*	*Si*	*Si*	*Si*	*Si*	*Si*	*Si*			
*Calomera littoralis aulicoides*													
											
					*Si*	*Si*							
											
*Calomera littoralis winkleri*													
											
*Cylindera contorta valdenbergi*													
											
											
											
*Cicindela javeti azari*													
											
*Myriochila melancholica melancholica*													
											
											
											
*Habrodera nilotica nilotica*				*Si*									*All-yaer*
											
*Calomera aulica aulica*				*(Si)*	*(Si)*		*(Si)*	*(Si)*					
											
					*Si*				*(Si)*	*Si*			
		*(Si)*	*(Si)*	*(Si)*	*(Si)*	*(Si)*	*(Si)*	*(Si)*	*(Si)*	*(Si)*			
*Lophyra flexuosa flexuosa*					*(Si)*								
											
					*(Si)*	*(Si)*							
											
	I	II	III	IV	V	VI	VII	VIII	IX	X	XI	XII	

Notes. *Si* – records only on the Sinai Peninsula, (*Si*) – records including the Sinai Peninsula. The density of the grey color corresponds with the frequency of the records of species (subspecies):

**Table:** 

	1–3		4–6		7–9		10–12		13–15		16–18

It should be noted that the period of activity of some studied species does not correspond with the data of previous studies in Israel (Nussbaum 1987) and on the Sinai Peninsula (Alfieri 1975; Abdel-Dayem et al. 2003), as well as in the other parts of the distribution area (Jaskuła and Rewicz 2015; Jaskuła et al. 2015). For example, the activity of *Calomera aulica aulica*, *Cicindela littoralis aulicoides*, *Cephalota zarudniana vartianorum*, *Cylindera contorta valdenbergi* and *Grammognatha euphratica euphratica* start one-two months earlier, while the activity of *Calomera aulica aulica*, *Cicindela littoralis aulicoides*, *Myriochila melancholica melancholica* and *Grammognatha euphratica euphratica* finish one-three, and in the case with *Lophyra flexuosa flexuosa* even six months later comparing with the data of Nussbaum (1987). On the other hand, Nussbaum (1987) indicated longer period of activity of *Calomera littoralis winkleri* and *Cephalota tibialis tibialis* as well as the later finish of the activity of *Cylindera contorta valdenbergi* and *Cicindela javeti azari* (Table 2).

Similarly, the periods of activity of *Calomera aulica aulica*, *Lophyra flexuosa flexuosa* and *Myriochila melancholica melancholica* in the central and southern Levant as well as on the Sinai Peninsula are appreciably longer than in the Maghreb region. So, in Tunisia *Calomera aulica aulica* records only in June and July (Jaskuła and Rewicz 2015), while in Israel it active from March to December and on the Sinai Peninsula from February to October (Table 2). Both in Tunisia and Morocco the period of activity of *Lophyra flexuosa flexuosa* lasts from March-April to July (Jaskuła and Rewicz 2015; Jaskuła et al. 2015) but in Israel it continues from February to December (Table 2).

On the contrary, in Tunisia the activity of *Grammognatha euphratica euphratica* begins in March and ends in July (Jaskuła and Rewicz 2015) that is similar with the period of activity in Israel and on the Sinai Peninsula (Table 2), while in Morocco it takes only three months from June to August (Jaskuła et al. 2015). The same situation is observed for different subspecies of *Cephalota littorea* (Forskål, 1775) as well as *Cicindela littoralis*. In Tunisia *Cephalota littorea gouditii* (Dejean, 1829) is active from May to October (Jaskuła and Rewicz 2015) while the period of activity of *Cephalota littorea littorea* on the Sinai Peninsula lasts from May to September (Table 2). The activity of *Cicindela littoralis littoralis* in Morocco is observed from April to October (Jaskuła et al. 2015) and in Tunisia from March to August (Jaskuła and Rewicz 2015), while the activity of *Cicindela littoralis aulicoides* in Israel and on the Sinai Peninsula as well as *Calomera littoralis winkleri* in Israel occurs from February to October and from February to November, respectively (Table 2).

However, we must remember that the obtained data are compilative. The differences in the time and the density of sampling, the collection technics as well as the frequency of visit of the particular localities and habitats could really distort the real pattern.

## Faunogenesis

The tiger beetle fauna of Israel as well as the Levant as a whole is complex. In geological time these areas were settled by species from different Mediterranean, African and Asiatic regions.

Unfortunately, the information about fossil Cicindelinae is extremely scant (Nagano et al. 1982). At present time South American *Oxycheilopsis cretacicus* Cassola & Werner, 2004 (Lower Cretaceous *ca.* 125 Ma) is the oldest known fossil tiger beetle (Cassola and Werner 2004). Three samples of fossil cicindelids are known from the northern Europe Baltic Amber (Oligocene *ca.* 23-34 Ma). Despite the identification ambiguity of the species, the genera were interpreted as the recent ones (Nagano et al. 1982; Röschmann 1999) as most known fossil Carabidae and other Coleoptera (Alekseev 2013). All other fossil records of the tiger beetles from the Europe and northern America (USA and Canada) are dated from the Quaternary period from Pleistocene to Holocene, and all other species are interpreted as recent (Nagano et al. 1982).

By analogy with other groups of carabid beetles (Kataev 1984, 2011; Casale and Vigna Taglianti 1999; Ruiz et al. 2012), we can assume that the genesis of the ancestral taxa of most recent cicindelids in the Mediterranean region began in late Paleogene – early Neogene (on the border of Oligocene – Miocene). According to data of DNA analysis the divergence processes of taxa of subtribe Cicindelina began *ca.* 15-25 Ma with most intensity between 2–10 Ma (Barraclough and Vogler 2002; Pons et al. 2004; Tsuji et al. 2015). For example, the diversification of the species within *Cicindela hybrida* group started *ca.* 2 Ma (Cardoso and Vogler 2005), while the separation of the genus *Cosmodela* Rivalier, 1961 from other Cicindelinae took place *ca.* 2.2–5 Ma (López-López et al. 2015; Tsuji et al. 2015). Based on the fossil material we could be argued that at least 60,000–70,000 yrs. BP the recent species of tiger beetles were already presented both in the North America and in the Eurasia (Nagano et al. 1982).

The continental drift of the Arabian and Anatolian Plates, their collision and, as the result, closing the Neotethys Ocean during Oligocene-Miocene were the most important processes forming the Mediterranean Sea and the genesis of the terrestrial Mediterranean fauna. The Eurasian-African land-bridge formed during late Burdigalian – middle Serravallian *ca.* 12.5–18 Ma (Rögl 1998) initiated the species change/exchange between the Europe, Asia and Africa (Koufos et al. 2005). The territory of the Sinai Peninsula and the Levant free from the sea formed the first transit corridor. However, it was interrupted at least twice in Langhian (*ca.* 16–16.4 Ma) and in early Serravallian (*ca.* 13–13.3 Ma), while in Tortonian (*ca.* 11.6 Ma) the final connection of Arabian and Anatolian plates and isolation of the Mediterranean Sea took place (Rögl 1998, 1999). Because the Central and Southern Levant as well as the Sinai Peninsula were the part of the Arabian plate connected with the African continent (Rögl 1998; Popov et al. 2004; Robertson et al. 2012; Berra and Angiolini 2014) the African species *Grammognatha euphratica*, *Habrodera nilotica*, *Myriochila melancholica* and *Lophyra flexuosa* could have colonized these territories before the other species.

The sharp decrease of the level of the Mediterranean Sea in Messinian (*ca.* 5.5–6 Ma) caused the formation of both numerous shallow enclosed saline basins and the land-bridges between Southern Europe and Northern Africa (Rögl and Steininger 1983). In our opinion during this time the active divergence and dispersion of such halophilic genera as *Cephalota*, *Calomera* and *Hypaetha* as well as the species of the subgenus *Eugrapha* occurred. All of them are arisen in the saline landscapes along the seashores of Para- and Neotethys in the Southern Russland as well as Central and Western Asia. From these regions the ancestors of the recent taxa probably dispersed through the Middle East, Arabian Peninsula and Anatolia to the Levant and the Sinai Peninsula, and some of them to Northern Africa. The second stream of the migration was possible along the Mediterranean coast of Southern Europe. Following this some species reached the Iberian Peninsula, and then the western regions of Northern Africa. In contrast *Grammognatha euphratica*, *Myriochila melancholica*, *Lophyra flexuosa* could be populated Southern Europe (Garcia-Reina et al. 2014), Western and Central Asia as well as Sind and some regions of South-Eastern Asia. Finally, possible during the last Glacial Period, the ancestors of *Cicindela javeti* and *Cicindela herbacea* dispersed into the Levant from the Anatolia, a region characterized by a higher level of diversity of the species of the *Cicindela campestris* group (Cassola 1999; Franzen 2007; Deuve 2011, 2012; our unpublished data).

This proposed version of the biogeographical genesis of the fauna of tiger beetles of the Levant should be considered an initial hypothesis. Molecular analysis and more detailed paleontologic information are necessary to robustly reject or validate it.

## Supplementary Material

XML Treatment for Calomera aulica aulica

XML Treatment for Calomera littoralis aulicoides

XML Treatment for Calomera littoralis winkleri

XML Treatment for Cephalota (Taenidia) zarudniana vartianorum

XML Treatment for Cephalota (Taenidia) littorea littorea

XML Treatment for Cephalota (Taenidia) tibialis tibialis

XML Treatment for Cicindela (s. str.) javeti azari

XML Treatment for Cicindela (s. str.) herbacea herbacea

XML Treatment for Cylindera (Eugrapha) contorta valdenbergi

XML Treatment for Habrodera nilotica nilotica

XML Treatment for Hypaetha singularis

XML Treatment for Lophyra (s. str.) flexuosa flexuosa

XML Treatment for Myriochila (s. str.) melancholica melancholica

XML Treatment for Grammognatha euphratica euphratica
